# Rational Design of Novel Isosteviol-Derived Factor Xa Inhibitors Using Integrated QSAR, Molecular Docking, Molecular Dynamics, and MM/GBSA Analyses

**DOI:** 10.3390/biology15141149

**Published:** 2026-07-14

**Authors:** Paweł Gordon, Łukasz Szeleszczuk, Małgorzata Lasota, Dariusz Maciej Pisklak, Marcin Gackowski

**Affiliations:** 1 University of Health Sciences in Bydgoszcz, Jagiellońska 4 Str., 85-067 Bydgoszcz, Poland; p.gordon@wsnoz.edu.pl; 2 Department of Organic and Physical Chemistry, Medical University of Warsaw, 1 Banacha Str., 02-097 Warsaw, Poland; lukasz.szeleszczuk@wum.edu.pl; 3 Center for Biomedicine and Interdisciplinary Sciences, Faculty of Medicine, Jagiellonian University Medical College, 16 Grzegórzecka Str., 31-531 Krakow, Poland; malgorzata.lasota@uj.edu.pl; 4 Department of Toxicology and Bromatology, Faculty of Pharmacy, L. Rydygier Collegium Medicum in Bydgoszcz, Nicolaus Copernicus University in Torun, A. Jurasza 2 Str., 85-089 Bydgoszcz, Poland; marcin.gackowski@cm.umk.pl

**Keywords:** isosteviol, factor Xa inhibitors, QSAR, MARSplines, molecular docking, molecular dynamics, MM/GBSA, Dragon descriptors, anticoagulant agents, ADMET, SwissADME, pkCSM, drug-likeness

## Abstract

Factor Xa (FXa) remains an important target in the development of anticoagulant and antithrombotic agents. In this study, twenty isosteviol-derived oxime ether analogs previously reported as FXa inhibitors were used to develop a predictive QSAR model. The compounds were geometry-optimized at the B3LYP/6-311++G(d,p) level, and Dragon molecular descriptors were calculated from the optimized structures. After descriptor filtering, Random Forest-based supervised preselection and correlation-based pruning were applied. Several models of increasing complexity were evaluated, including multiple linear regression, additive MARSplines, and constrained second-order MARSplines models. The final model employed four active basis functions involving R6p+, C-025, ATSC7e, and Mor31p and demonstrated excellent calibration and cross-validated predictive ability (R^2^ = 0.929 and Q^2^_LOO = 0.865). Based on this model, twenty new isosteviol-derived analogs were designed and their activities were predicted after DFT optimization and descriptor calculation. Eight representative compounds were subsequently subjected to molecular docking against human factor Xa (PDB ID: 2P16), molecular dynamics simulations, MM/GBSA binding free energy calculations, and preliminary SwissADME/pkCSM profiling. Docking protocol validation yielded a redocking RMSD of 0.953 Å. Although ISV-M20 was the highest-ranked compound according to the QSAR model, subsequent receptor-based analyses identified ISV-M19, ISV-M04, and ISV-M06 as the derivatives with the most favorable combination of structural stability, persistent protein–ligand interactions, and binding free energies. The ADMET/toxicity screen further refined this prioritization: ISV-M19, ISV-M04, and ISV-M06 showed the most favorable preliminary toxicity balance among the prioritized derivatives, whereas ISV-M20 displayed additional developability liabilities, including very high lipophilicity, poor predicted solubility, P-gp substrate status, and a predicted hERG II alert. Overall, the results demonstrate that integrating interpretable QSAR modeling with receptor-based simulations and early ADMET/toxicity filtering provides a more balanced strategy for the rational design and prioritization of novel isosteviol-derived FXa inhibitors than any single computational approach alone.

## Simple Summary

Blood clot formation plays a central role in many cardiovascular diseases, and factor Xa is one of the key enzymes regulating coagulation. In this study, isosteviol-derived factor Xa inhibitor candidates were analyzed using an integrated computational workflow combining quantum-chemical calculations, molecular descriptors, QSAR modeling, molecular docking, molecular dynamics simulations, MM/GBSA binding free energy calculations, and preliminary ADMET/toxicity prediction. A constrained second-order MARSplines model was developed from a congeneric set of known isosteviol-derived oxime ether inhibitors and was then used to design twenty new analogs differing only in the terminal aromatic fragment. Although the QSAR model identified ISV-M20 as the most promising in-domain candidate, receptor-based analyses indicated that ISV-M19, ISV-M04, and ISV-M06 exhibited a more favorable combination of binding stability, persistent interactions within the factor Xa active site, and binding free energies. Preliminary SwissADME and pkCSM profiling further supported this integrated prioritization by indicating a cleaner predicted toxicity balance for ISV-M19, ISV-M04, and ISV-M06, whereas ISV-M20 showed additional developability concerns despite its high QSAR-predicted activity. Overall, the study shows that ligand-based QSAR predictions should be complemented by receptor-based and ADMET-aware filters when prioritizing new natural-product-derived factor Xa inhibitor candidates.

## 1. Introduction

Thromboembolic diseases remain a major clinical problem because pathological clot formation underlies or complicates several high-burden cardiovascular conditions, including venous thromboembolism, pulmonary embolism, ischemic stroke, and atrial fibrillation-related systemic embolism [1,2,3,4]. Activated coagulation factor X (factor Xa, FXa) occupies a strategic position at the convergence of the intrinsic and extrinsic coagulation pathways because it catalyzes prothrombin-to-thrombin conversion within the prothrombinase complex. Direct oral FXa inhibitors, including rivaroxaban, apixaban, edoxaban, and betrixaban, provide rapid and predictable anticoagulation [5], but bleeding risk, patient-specific dosing, drug–drug interactions, renal impairment, perioperative management, and reversal of life-threatening bleeding remain clinically demanding [2,3,4,5,6]. These limitations justify continued exploration of alternative FXa inhibitor scaffolds and improved structure–activity understanding.

Structurally diverse direct FXa inhibitors differ in their peripheral scaffolds but generally target the canonical orthosteric active-site cleft of FXa and exploit the S1/S4 recognition environment. In the apixaban-bound crystal structure used in the present work, the ligand occupies the established inhibitor-binding pocket and engages an active-site architecture defined by residues such as Tyr99, Phe174, Trp215, Asp189, and Gly216. This experimentally defined binding mode provides a suitable structural reference for docking natural-product-derived FXa inhibitor candidates into the validated direct-inhibitor binding pocket.

Natural products and semi-synthetic natural-product derivatives remain valuable sources of molecular diversity for medicinal chemistry because their rigid three-dimensional architectures and functionally dense scaffolds can provide binding modes that are not easily obtained from flat aromatic screening libraries [7,8]. Isosteviol, a tetracyclic diterpenoid obtained from stevioside, combines a compact hydrophobic skeleton with modifiable functional groups and has been investigated as a starting point for biologically active derivatives [9]. The relevance of this scaffold to anticoagulant drug discovery was strengthened by Chen et al., who reported isosteviol-derived antithrombotic agents and identified oxime ether analogs with measurable human FXa inhibitory activity, including a sulfur-containing bicyclic heteroaryl derivative with submicromolar potency [10]. Subsequent in silico studies further supported the applicability of QSAR, molecular docking, molecular dynamics, and ADMET prediction to isosteviol-derived anticoagulant candidates [11,12,13], but the oxime ether 6ra-6rt series has not yet been fully exploited as a compact congeneric dataset for interpretable descriptor-based modeling and model-guided analog design.

Quantitative structure–activity relationship (QSAR) modeling can translate small experimental datasets into testable medicinal-chemistry hypotheses, provided that model fit is interpreted together with validation, parsimony, y-randomization, and applicability-domain analysis [14,15]. Among nonlinear regression techniques, multivariate adaptive regression splines (MARSplines) are particularly useful because they can capture threshold-type and interaction-dependent structure–activity relationships while retaining an explicit mathematical form [16,17]. The aim of the present work was therefore to develop a compact, internally validated, and interpretable MARSplines QSAR model for FXa inhibition in a congeneric series of isosteviol-derived oxime ethers and to use this model as a rational design tool. The workflow combined DFT-based geometry optimization, Dragon descriptor calculation, supervised descriptor reduction, constrained MARSplines modeling, applicability-domain analysis, model-guided design of twenty new fixed-core R2 analogs, docking, molecular dynamics, MM/GBSA calculations, and preliminary ADMET/toxicity assessment.

## 2. Materials and Methods

### 2.1. Dataset Preparation

The QSAR dataset consisted of twenty isosteviol-derived oxime ether derivatives previously reported as human FXa inhibitors [10]. The compounds corresponded to the 6ra-6rt series and were recoded as i_a–i_t for the purpose of this computational study. The experimentally determined FXa inhibitory activity was expressed as pKi_FXa and used as the dependent variable in QSAR modeling. The pKi_FXa values were calculated from Ki values expressed in micromolar according to Equation (1):

pKi_FXa = 6 − log10(Ki_FXa [µM]).(1)

This transformation was applied so that higher pKi_FXa values corresponded to higher inhibitory potency.

The complete source dataset, including the general scaffold, original compound codes, internal QSAR codes, R2 substituents, experimental Ki_FXa values, and transformed pKi_FXa endpoints, is summarized in Table 1.

### 2.2. Geometry Optimization

Three-dimensional structures of the investigated compounds were prepared and geometry-optimized prior to descriptor calculation. All quantum-chemical calculations were performed using Gaussian 16, Revision C.01 [18], and initial molecular geometries were generated and visually inspected using GaussView 6 [19]. Geometry optimization was carried out using the B3LYP hybrid density functional [20,21,22] with the 6-311++G(d,p) basis set [23,24,25,26]. Frequency calculations were performed at the same level of theory to confirm that the optimized structures corresponded to stationary points on the potential-energy surface and to exclude imaginary frequencies. The optimized geometries were subsequently used for molecular descriptor calculation. This geometry-optimization and descriptor-generation workflow follows the general strategy previously applied in related QSAR modeling of small-molecule enzyme inhibitors [14].

### 2.3. Molecular Descriptor Calculation and Initial Filtering

Molecular descriptors were calculated from the optimized geometries using Dragon software, version 7 [27]. The obtained descriptors included constitutional, topological, geometrical, autocorrelation, GETAWAY, 3D-MoRSE, RDF, atom-centered fragment, and other descriptor classes. Before QSAR modeling, the descriptor matrix was subjected to objective technical filtering. Descriptors with missing values, constant or near-constant values, very low standard deviation, and excessive pairwise intercorrelation were removed. This step was applied to reduce descriptor redundancy and eliminate variables unsuitable for regression modeling.

### 2.4. Supervised Descriptor Reduction

After technical filtering, supervised descriptor preselection was performed using Random Forest regression with pKi_FXa as the response variable [28]. Variable importance ranking was used to identify descriptors most strongly associated with FXa inhibitory activity. The resulting candidate descriptor pool was further pruned by removing highly intercorrelated variables. Because the dataset contained only twenty compounds, model complexity was deliberately limited. The final models were restricted to a maximum of four active descriptors or model terms, maintaining a conservative relationship between the number of compounds and the number of variables included in the QSAR model.

### 2.5. MLR and MARSplines Modeling

Multiple linear regression (MLR) models were initially developed as linear baseline models. Candidate 3-descriptor and 4-descriptor models were compared using calibration and cross-validation statistics. These models were used as reference models for assessing whether nonlinear MARSplines regression offered a meaningful improvement in predictive performance. The regression procedures were implemented using TIBCO Statistica software, version 13.3.0 (TIBCO Software Inc., Palo Alto, CA, USA) [29].

MARSplines regression was applied to model potential nonlinear relationships between molecular descriptors and FXa inhibitory activity [16,17]. The method constructs piecewise linear basis functions of the general form h(x) = max(0,x), where the position of the knot defines the point at which a descriptor begins to contribute to the model. Both additive and second-order MARSplines models were evaluated. The additive model did not include interaction terms, whereas the second-order model allowed pairwise interactions between basis functions. To reduce the risk of overfitting, the number of active basis functions was restricted to four. The final model was selected based on cross-validated predictive performance, calibration error, parsimony, y-randomization, and applicability-domain analysis.

### 2.6. Model Validation

The developed models were validated according to commonly accepted QSAR validation principles, including the OECD recommendations, internal cross-validation, y-randomization, and applicability-domain assessment [15,30,31,32,33,34]. Goodness-of-fit was assessed using R^2^, adjusted R^2^, RMSEC, and calibration MAE. Predictive ability was assessed using leave-one-out cross-validation, expressed as Q^2^_LOO, PRESS, RMSECV_LOO, and MAE_LOO. To further assess model robustness, repeated 5-fold cross-validation was performed. Mean Q^2^, standard deviation of Q^2^, mean RMSE, and mean MAE were calculated to assess the stability of predictive performance.

Y-randomization was performed to evaluate whether the obtained model could have arisen by chance and to reduce the risk of interpreting accidental descriptor-response relationships as meaningful SAR patterns [30,31,32,33]. In this procedure, the response variable was randomly permuted 1000 times, while the descriptor matrix was kept unchanged. For each permutation, the same fixed model form was evaluated, and randomized R^2^ and Q^2^_LOO values were compared with those of the original model. Multicollinearity was assessed using the variance inflation factor (VIF) and pairwise correlations between the final basis functions.

### 2.7. Applicability Domain

The applicability domain was assessed using leverage analysis and standardized residuals, in line with established QSAR validation and applicability-domain principles [15,32,34]. The warning leverage threshold was calculated according to Equation (2):

h* = 3(*k* + 1)/*n*. (2)

In this equation, *k* is the number of active model terms and *n* is the number of compounds in the dataset. For the final model, *k* = 4 and *n* = 20, resulting in h* = 0.75. Compounds with leverage values above h* were considered structurally influential. Compounds with standardized residuals exceeding |3| were considered response outliers. The model applicability domain was additionally characterized by the observed training-set ranges of the four descriptors retained in the final model: R6p+, C-025, ATSC7e, and Mor31p.

### 2.8. Model-Guided Design of New Analogs, Gaussian Input Preparation, and Downstream Prioritization

The final constrained second-order MARSplines model was used to guide the design of a focused series of new isosteviol-derived analogs. To preserve the structural coherence of the training set and to reduce the risk of extrapolation outside the applicability domain, the tetracyclic isosteviol scaffold, the ethyl ester moiety, and the C16 oxime ether linker were kept unchanged. Structural modifications were restricted to the terminal R2 substituent in the O-CH2-CH=CH-R2 fragment.

The restriction to the terminal R2 fragment was therefore not arbitrary. It reflected the structure of the available experimental SAR dataset, in which the common isosteviol oxime ether framework was preserved and activity differences were driven mainly by changes in the terminal aromatic or heteroaromatic substituent. Keeping the scaffold, ester group, and oxime ether linker unchanged allowed the newly designed analogs to remain chemically comparable with the training compounds and reduced the risk of applying the MARSplines model outside its descriptor-defined applicability domain.

The design strategy was based on the descriptor-domain requirements of the final model and on the structure–activity pattern observed in the training series. Particular attention was paid to analogs related to the most active compound, i_m, and to small, chemically conservative modifications of benzothiophene, benzothiazole, benzofuran, N-methylindole, and selected substituted phenyl fragments. The proposed compounds were intended as a first focused design panel rather than as experimentally validated inhibitors.

For each proposed molecule, a starting three-dimensional structure was prepared as a Gaussian input file. All files were generated as neutral singlet structures and used the same route section as the training compounds, #p B3LYP/6-311++G(d,p) Opt Freq. The designed molecules were subsequently geometry-optimized and their optimized structures were used to calculate Dragon descriptors. The final QSAR model was applied after verifying the availability of R6p+, C-025, ATSC7e, and Mor31p and checking whether these descriptors remained within or close to the training-set ranges defining the applicability domain.

Selection of compounds for the next computational stage was performed using a hierarchical prioritization scheme. First, practical descriptor-domain status was used as a mandatory filter for the primary selection. Second, in-domain compounds were ranked according to the predicted Ki_FXa values obtained from the final MARSplines model. Third, chemical interpretability and representation of the designed substitution space were considered. Accordingly, the five highest-ranked in-domain compounds, ISV-M20, ISV-M17, ISV-M18, ISV-M16, and ISV-M19, were selected as the primary subset for subsequent molecular docking, molecular dynamics, and MM/GBSA calculations. In parallel, ISV-M04, ISV-M06, and ISV-M09 were retained as exploratory docking comparators because they showed favorable predicted activity but included at least one descriptor-domain violation. These outside-domain analogs were not treated as primary MD/MMGBSA candidates unless docking poses and interaction profiles provided additional justification.

### 2.9. Molecular Docking Calculations

#### 2.9.1. Ligand Preparation

The structures of the investigated isosteviol derivatives (ISV-M04, ISV-M06, ISV-M09, ISV-M16, ISV-M17, ISV-M18, ISV-M19, and ISV-M20) were constructed using Schrödinger Maestro 2023-3 (Schrödinger, LLC, New York, NY, USA). Ligand geometries were prepared and optimized using the LigPrep module with the OPLS4 force field. Possible ionization states were generated at physiological pH (7.4 ± 0.5), and low-energy conformations were retained for subsequent docking studies.

#### 2.9.2. Protein Preparation

The crystal structure of human coagulation factor Xa (FXa) in complex with apixaban (PDB ID: 2P16) was retrieved from the Protein Data Bank. Protein preparation was performed using the Protein Preparation Wizard implemented in Schrödinger Maestro. Bond orders were assigned, hydrogen atoms were added, missing side chains were completed where necessary, and protonation states were optimized at physiological pH. Water molecules located outside the active site were removed. The prepared structure was subsequently subjected to restrained energy minimization using the OPLS4 force field.

#### 2.9.3. Docking Protocol Validation

To validate the docking procedure, the co-crystallized ligand apixaban was extracted from the crystal structure and subsequently redocked into the FXa active site using the same protocol applied to the investigated compounds. The resulting docking pose reproduced the experimental binding mode with a root-mean-square deviation (RMSD) of 0.953 Å, confirming the reliability of the docking methodology.

#### 2.9.4. Molecular Docking Procedure

Receptor grids were generated using the coordinates of the co-crystallized apixaban molecule as the center of the binding site. Molecular docking calculations were performed using the Glide Extra Precision (XP) protocol implemented in Schrödinger Maestro. Default Glide XP settings were employed. The best-ranked pose for each ligand was selected based on GlideScore values and visual inspection of ligand orientation within the active site. Protein–ligand interactions, including hydrogen bonds, hydrophobic contacts, π-π interactions, and other stabilizing contacts, were analyzed using Maestro visualization tools. For receptor grid generation, the docking grid was centered on the centroid of the co-crystallized ligand in the factor Xa active site. The default Glide receptor grid dimensions were used, with an inner (ligand diameter midpoint) box of 10 × 10 × 10 Å and an outer (bounding) box of 30 × 30 × 30 Å, ensuring complete coverage of the binding pocket. Crystallographic water molecules not directly involved in ligand recognition were removed prior to grid generation. No positional, hydrogen-bonding, or metal coordination constraints were applied during docking. Docking calculations were performed using the Glide XP protocol with default sampling settings and without enhanced sampling options. The docking analysis focused on the canonical S1 and S4 subsites of the factor Xa active site, including the key residues involved in ligand recognition, namely Tyr99, Asp189, Phe174, Trp215, and Gly216. No additional allosteric or alternative binding pockets were explored, as the experimental endpoint used for QSAR model development was inhibition of human factor Xa, and the objective of the structure-based stage was to evaluate whether the designed analogs could adopt stable binding modes within the established active site of direct factor Xa inhibitors.

### 2.10. Molecular Dynamics Simulations

The docked complexes of FXa with apixaban and the investigated isosteviol derivatives were subjected to molecular dynamics (MD) simulations using the Desmond module implemented in Schrödinger Maestro 2023-3. Each complex was solvated in an orthorhombic simulation box filled with TIP3P water molecules, maintaining a 10 Å buffer distance from the protein surface. Counterions were added to neutralize the systems, and physiological ionic strength was achieved by addition of 0.15 M NaCl.

Prior to production runs, all systems were relaxed using the default Desmond relaxation protocol consisting of energy minimization and gradual equilibration. Production simulations were performed under NPT conditions at 300 K and 1 atm for 100 ns using the Nose-Hoover thermostat and Martyna-Tobias-Klein barostat. The integration time step was set to 2.0 fs, and trajectory frames were recorded every 100 ps.

To assess the reproducibility of the simulation results, two additional independent 100 ns molecular dynamics simulations were performed for the three top-performing ligands (ISV-M19, ISV-M04, and ISV-M06). Each replicate was initiated from the same docked complex but with independently randomized initial atomic velocities while all remaining simulation parameters were kept identical.

For the four key complexes selected for detailed receptor-based evaluation, namely FXa–apixaban, FXa–ISV-M19, FXa–ISV-M04, and FXa–ISV-M06, the production MD simulations were extended to 200 ns (Figure S3). These extended simulations were performed using the same system preparation, equilibration protocol, force field, solvent model, temperature, pressure, and trajectory-recording settings as described above. The 200 ns trajectories were subsequently used for the final RMSD, RMSF, protein–ligand contact, ligand-property, and MM/GBSA analyses reported in the revised manuscript.

#### 2.10.1. Trajectory Analysis

Simulation trajectories were analyzed using the Simulation Interaction Diagram tool implemented in Desmond. Structural stability was assessed by monitoring the root-mean-square deviation (RMSD) of protein backbone atoms and ligand heavy atoms. Protein–ligand interaction profiles, including hydrogen bonds, hydrophobic contacts, ionic interactions, and water bridges, were evaluated throughout the simulation. Representative interaction patterns and contact occupancies were used to characterize the stability of ligand binding within the FXa active site.

#### 2.10.2. MM/GBSA Binding Free Energy Calculations

The binding free energies of the investigated ligand–FXa complexes were estimated using the Molecular Mechanics Generalized Born Surface Area (MM/GBSA) approach implemented in the Prime module of Schrödinger Maestro 2023-3. MM/GBSA calculations were performed using representative frames extracted from the molecular dynamics trajectories.

Trajectory frames collected during the final 20 ns of each simulation were used for energy evaluation to ensure that only equilibrated conformations contributed to the calculations. The VSGB 2.1 implicit solvent model and the OPLS4 force field were employed. For each complex, the binding free energy (ΔG_bind) was calculated according to Equation (3):

ΔG_bind_ = G_complex_ − (G_protein_ + G_ligand_) (3)

where G_complex_, G_protein_, and G_ligand_ represent the free energies of the protein–ligand complex, the isolated protein, and the isolated ligand, respectively.

The reported MM/GBSA values correspond to the average binding free energy obtained from the analyzed trajectory frames and were used to compare the relative binding affinities and stability of the investigated isosteviol derivatives toward factor Xa.

Although MM-PBSA is a valuable alternative endpoint for binding free-energy estimation, the present study used Prime MM/GBSA with the OPLS4 force field and VSGB 2.1 solvation model to maintain methodological consistency with the Schrödinger docking and MD workflow. The resulting binding energies were interpreted only as approximate comparative estimates within a congeneric ligand series, not as absolute experimental affinities. The methodological rationale for combining MD simulations with MM/GBSA-based binding-free-energy assessment is consistent with recent long-timescale simulation studies using MM/GBSA for in silico ligand prioritization [35].

#### 2.10.3. Statistical Analysis of MD Descriptors

For exploratory statistical comparison of the MD-derived descriptors, the equilibrated portions of the 200 ns trajectories were divided into 5 ns blocks to reduce the influence of frame-to-frame autocorrelation. Block-averaged values calculated over the 150–200 ns interval were then compared between FXa–apixaban and the prioritized ISV complexes using Welch’s *t*-test. Residue-wise Cα RMSF values for the same set of key binding-site residues were compared using paired *t*-tests. All statistical analyses were performed using TIBCO Statistica software, version 13.3.0 (TIBCO Software Inc., Palo Alto, CA, USA). The statistical analysis was considered exploratory and was used only to support the interpretation of trajectory-derived stability descriptors. Docking scores were not subjected to statistical testing because a single best-ranked docking score was obtained for each ligand and no variance estimate was available.

### 2.11. In Silico ADMET and Toxicity Prediction

Preliminary ADMET, drug-likeness, and toxicity profiling was performed for the newly designed ISV-M01-ISV-M20 analogs using SwissADME (version 14.9.29) and pkCSM (version 2026) [36,37]. SwissADME was used to estimate physicochemical properties, lipophilicity, water solubility, gastrointestinal absorption, blood–brain barrier permeation, P-glycoprotein substrate status, drug-likeness filters, medicinal-chemistry alerts, bioavailability score, and synthetic accessibility. Because SwissADME does not provide a full toxicological profile, pkCSM was additionally used to predict AMES toxicity, maximum tolerated human dose, hERG I and hERG II inhibition, acute and chronic oral rat toxicity, hepatotoxicity, skin sensitization, T. pyriformis toxicity, and minnow toxicity. These predictions were interpreted as an early-stage developability screen complementary to the QSAR, docking, molecular-dynamics, and MM/GBSA analyses, rather than as a substitute for experimental pharmacokinetic or toxicological evaluation.

## 3. Results

### 3.1. QSAR Dataset and Endpoint Definition

The QSAR analysis was performed for a congeneric set of twenty isosteviol-derived oxime ether derivatives previously reported as human FXa inhibitors. The compounds corresponded to the 6ra-6rt series and were recoded in the present study as i_a-i_t for consistency with the computational workflow. The FXa inhibitory activity was expressed as pKi_FXa and used as the dependent variable in regression modeling. The analyzed series covered a broad inhibitory activity range, from weakly active analogs with pKi_FXa values around 4.58 to the most potent compound i_m, with an observed pKi_FXa of approximately 6.68. This range was considered sufficient for developing a regression-based QSAR model within this structurally related series.

After geometry optimization and Dragon descriptor calculation, the initial descriptor pool was subjected to standard technical filtering. The filtered matrix was then subjected to supervised descriptor preselection based on Random Forest regression, followed by correlation-based pruning. Because of the limited dataset size, the final QSAR models were deliberately restricted to a maximum of four active terms, in line with the conservative requirement of maintaining an adequate compound-to-variable ratio.

### 3.2. Comparative Evaluation of Candidate QSAR Models

Several regression approaches of increasing complexity were evaluated to identify the most reliable predictive model. First, MLR models were developed as conservative linear baselines. The best 3-descriptor MLR model included MATS8i, Mor31p, and R3u and yielded R^2^ = 0.799 and Q^2^_LOO = 0.652. The best 4-descriptor MLR model, based on Mor31p, Mor31m, RGyr, and R6p+, provided R^2^ = 0.797 and Q^2^_LOO = 0.681. Although these models showed acceptable internal performance, their predictive ability remained moderate.

Therefore, MARSplines regression was further applied to account for possible nonlinear relationships between Dragon descriptors and FXa inhibitory activity. The additive MARSplines model without interaction terms markedly improved the model statistics, reaching R^2^ = 0.905 and Q^2^_LOO = 0.854. Finally, a constrained second-order MARSplines model was evaluated. This model allowed pairwise interactions between selected spline terms but retained the predefined limit of four active basis functions. The second-order model showed the best balance between goodness-of-fit, internal predictivity, and model parsimony, with R^2^ = 0.929, adjusted R^2^ = 0.910, Q^2^_LOO = 0.865, RMSECV_LOO = 0.180, and MAE_LOO = 0.139. It was therefore selected as the final predictive QSAR model (Table 2).

Model selection was based primarily on cross-validated performance, prediction error, robustness, parsimony, and applicability-domain diagnostics rather than calibration R^2^ alone. More complex spline variants were not retained because the improvement in apparent fit was considered insufficient to justify the increased risk of overfitting.

### 3.3. Final Constrained Second-Order MARSplines Model

The final QSAR model was a constrained second-order MARSplines model composed of four active basis functions. The model retained four Dragon descriptors: R6p+, C-025, ATSC7e, and Mor31p. The resulting regression equation is presented as Equation (4):

(4) $$\begin{array}{l} \text{pKi\_FXa}=5.557080+678.580929\times h\left(R6p+-\;0.008\right)\times h\left(\mathrm{Mor}31p-0.871\right)\\ \text{-}\;1.648818\times h(C\text{-}025-1)\times h(1.136-\mathrm{Mor}31p)\\ \text{-}\;100.735200\times h(0.496-\mathrm{ATSC}7e)\times h(1.136-\mathrm{Mor}31p)\\ \text{-}\;8032.776775\times h(0.008-R6p+)\times h(0.547-\mathrm{ATSC}7e), \end{array}$$

where h(x) = max(0,x).

The model indicates that FXa inhibitory activity in the analyzed isosteviol series is governed by nonlinear interactions between descriptors related to polarizability-weighted 3D molecular geometry, electronegativity-weighted autocorrelation, 3D-MoRSE information, and atom-centered fragment characteristics. Importantly, the final model remained compact, with only four basis functions, despite the inclusion of second-order interaction terms (Table 3).

The positive coefficient of BF1 suggests that the simultaneous increase in R6p+ above the knot value of 0.008 and Mor31p above 0.871 contributes favorably to the predicted FXa inhibitory activity. Conversely, BF2-BF4 introduce negative contributions under specific descriptor conditions, indicating that unfavorable combinations of atom-centered fragments, electronegativity distribution, and low polarizability-weighted geometry may reduce the predicted activity.

From a chemical perspective, the retained descriptors indicate that FXa inhibition within this congeneric isosteviol oxime ether series is not governed by a single substituent property, but rather by the combined influence of the rigid hydrophobic isosteviol core and the electronic and spatial characteristics of the terminal R2 fragment. R6p+ and Mor31p encode three-dimensional geometry and polarizability-weighted molecular distribution, which is consistent with the need to maintain productive hydrophobic occupation of the FXa S1/S4 recognition environment by the bulky diterpenoid scaffold and terminal aromatic moiety. ATSC7e reflects electronegativity-weighted autocorrelation over a defined topological distance and therefore captures how heteroatoms, halogens, and electron-rich aromatic systems modulate the electronic pattern of the oxime ether side chain. C-025, as an atom-centered fragment descriptor, reflects the occurrence of specific carbon environments introduced by the terminal substituent. Therefore, the model suggests that favorable activity requires a balanced combination of molecular shape, polarizability, electronic distribution, and substituent-specific carbon environments rather than simple maximization of lipophilicity or aromatic bulk.

### 3.4. Model Fit and Internal Validation

The final second-order MARSplines model showed strong calibration performance. The coefficient of determination was R^2^ = 0.9286, while the adjusted R^2^ was 0.9095, indicating that the model explained most of the variance in the observed pKi_FXa values while maintaining acceptable complexity. The Pearson correlation coefficient between experimental and predicted values was 0.9636, and the concordance correlation coefficient was 0.9630. The calibration error was low, with RMSEC = 0.1308 and MAE = 0.0977 pKi units.

Leave-one-out cross-validation confirmed good internal predictive ability. The model yielded Q^2^_LOO = 0.8646, PRESS = 0.6487, RMSECV_LOO = 0.1801, and MAE_LOO = 0.1388. The model was also statistically significant, with F = 48.75 and *p* = 2.02 × 10^−8^. The maximum variance inflation factor was 1.1467, and the maximum absolute correlation between basis functions was 0.2602, indicating the absence of relevant multicollinearity.

A repeated 5-fold cross-validation procedure was additionally used as a robustness test. Across 500 repeated splits, the model retained stable predictive performance, with mean Q^2^ = 0.8363, SD Q^2^ = 0.0520, mean RMSE = 0.1958, and mean MAE = 0.1491. The 5th and 95th percentiles of Q^2^ were 0.7376 and 0.8978, respectively, confirming that the model did not depend on a single favorable data split (Table 4).

### 3.5. Predicted Versus Experimental Activity

**Figure 1:**
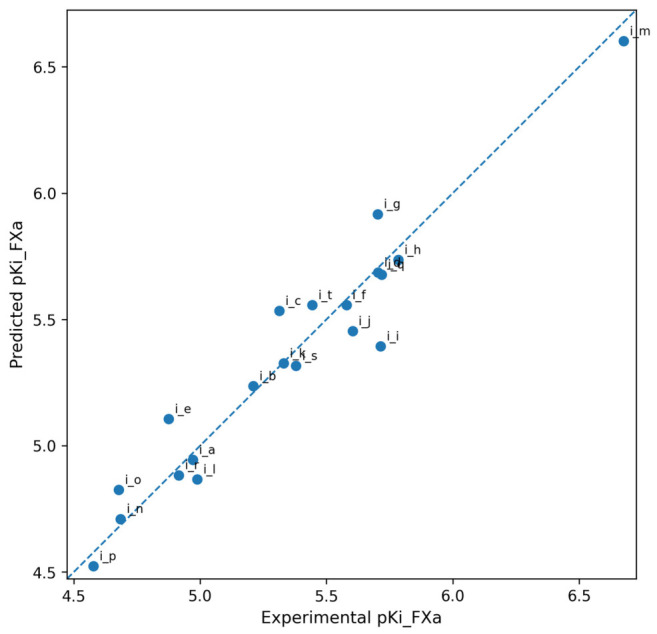
Experimental versus predicted pKi_FXa values for the final constrained second-order MARSplines model. The dashed line represents the ideal y = x relationship. Experimental FXa inhibitory activity was expressed as pKi_FXa and used as the dependent variable in QSAR modeling. Most compounds were located close to the identity line, supporting good agreement between experimental and calculated values. Compounds i_a and i_m showed high leverage in the applicability domain analysis, but neither behaved as a response outlier.

The graphical relationship between experimental and predicted pKi_FXa values is shown in Figure 1. Most compounds were located close to the identity line, confirming good agreement between experimental and predicted activity values. The most active compound, i_m, was also predicted accurately, despite its influential structural position in the dataset.

### 3.6. Y-Randomization

To verify that the final model did not result from chance correlation, a fixed-form y-randomization test was performed using 1000 random permutations of the dependent variable. None of the randomized models achieved an R^2^ or Q^2^_LOO equal to or greater than the observed model. The maximum randomized R^2^ was 0.7582, and the maximum randomized Q^2^_LOO was 0.5731, both clearly below the values obtained for the real model. The mean randomized Q^2^_LOO was −0.7334, further supporting the non-random character of the relationship captured by the final model (Table 5).

### 3.7. Applicability Domain

The applicability domain of the final model was evaluated using leverage analysis. For the present model, h* = 0.75. Two compounds, i_a and i_m, exceeded the warning leverage threshold and were therefore identified as structurally influential observations. However, no compound showed an absolute standardized residual greater than 3. Therefore, i_a and i_m were not considered response outliers. Compound i_m is particularly important because it is the most active compound in the dataset and was predicted well by the final model. Its high leverage should therefore be interpreted as reflecting its boundary position within the descriptor space rather than model failure.

The practical descriptor ranges defining the applicability domain are shown in Table 6 and Table 7. Future predictions for newly designed isosteviol analogs should be considered most reliable when their R6p+, C-025, ATSC7e, and Mor31p values remain within or close to the ranges observed for the training compounds.

### 3.8. Selection of the Final Predictive Model

The results indicate that FXa inhibitory activity in the analyzed isosteviol oxime ether series is better described by a compact nonlinear model than by a purely linear regression equation. The final constrained second-order MARSplines model provided the most favorable balance between model fit, internal predictivity, parsimony, and robustness. The model was supported by leave-one-out cross-validation, repeated 5-fold cross-validation, y-randomization, low multicollinearity, and applicability domain analysis. Therefore, this model was selected as the final predictive QSAR tool for prioritizing newly designed isosteviol-derived FXa inhibitors in the next stage of the study.

### 3.9. Model-Guided Design of Twenty New Isosteviol-Derived Analogs

After selection of the final MARSplines model, the quantitative structure–activity information was used to design a focused panel of twenty new isosteviol-derived oxime ether analogs. The design was deliberately conservative: the isosteviol scaffold, ethyl ester group, C16 oxime ether linkage, and allyl-type O-CH2-CH=CH-R2 chain were retained, while only the terminal R2 substituent was modified. This strategy was chosen to maximize the likelihood that the newly designed molecules would remain close to the applicability domain of the model. Consequently, the designed set should be interpreted as a focused R2-optimization panel rather than as a broad scaffold-hopping library.

The final model suggested that favorable predictions should be associated with structures capable of maintaining R6p+ and Mor31p within the upper part of the training-set range while avoiding combinations of low R6p+ and low ATSC7e, which would activate a strongly negative basis-function contribution. Therefore, the design focused primarily on benzothiophene-like and sulfur-containing bicyclic heteroaryl fragments related to the most active training compound i_m, supplemented with benzothiazole, benzofuran, N-methylindole, and selected substituted phenyl analogs to provide controlled structural diversity.

**Figure 2:**
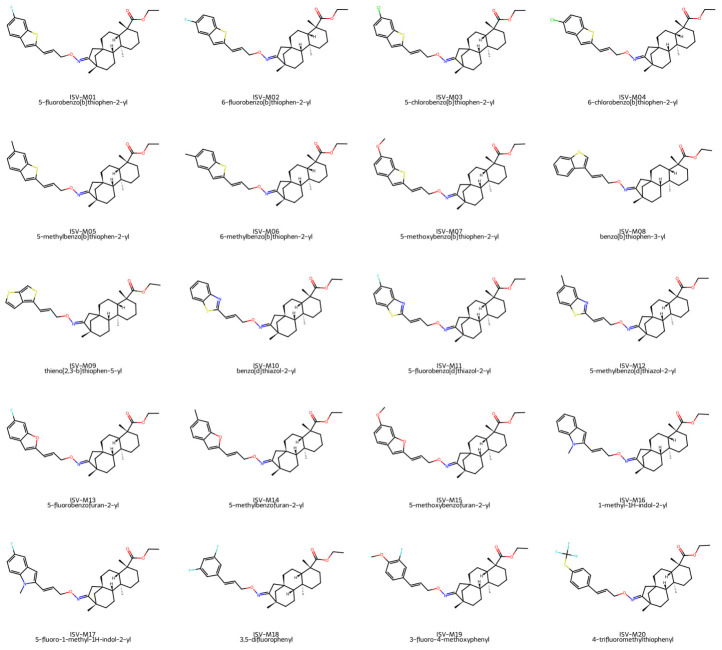
Proposed focused series of twenty newly designed isosteviol-derived oxime ether analogs (ISV-M01-ISV-M20). The common isosteviol scaffold and oxime ether linker were retained, whereas the terminal R2 substituent was modified to explore benzothiophene, benzothiazole, benzofuran, indole, and selected substituted phenyl fragments. These structures represent the first model-guided design panel and were subsequently subjected to DFT optimization, Dragon descriptor calculation, applicability-domain assessment, and QSAR-based activity prediction.

The proposed compounds are summarized in Table 8 and visualized in Figure 2. After Gaussian optimization and Dragon descriptor calculation, all four descriptors required by the final model were available for ISV-M01-ISV-M20, enabling direct calculation of predicted pKi_FXa and Ki_FXa values. Values marked with an asterisk include at least one descriptor-domain violation and should therefore be interpreted as extrapolative predictions.

Based on the calculated descriptor profile and final MARSplines predictions, ISV-M20 emerged as the most promising in-domain candidate, with a predicted pKi_FXa of 6.09 and an estimated Ki_FXa of 0.81 µM. Other in-domain candidates with comparatively favorable predicted values included ISV-M17, ISV-M18, ISV-M16, and ISV-M19. These five compounds were therefore selected as the primary subset for downstream molecular docking, molecular dynamics, and MM/GBSA calculations. This prioritization was not based solely on absolute predicted activity; in-domain status was treated as a mandatory first filter to avoid overinterpreting extrapolative QSAR predictions. ISV-M04, ISV-M06, and ISV-M09 also showed comparatively favorable predicted values, but their interpretation requires caution because at least one final-model descriptor was outside the training-set descriptor range. Although these compounds exhibited at least one descriptor-domain violation, they were retained for structure-based studies because of their favorable predicted activities and chemical relevance.

These predictions provide a quantitative basis for selecting compounds for the subsequent docking, molecular dynamics, MM/GBSA, SwissADME, and prospective experimental stages. The primary next-stage set comprises ISV-M20, ISV-M17, ISV-M18, ISV-M16, and ISV-M19. In addition, ISV-M04, ISV-M06, and ISV-M09 constitute an exploratory docking-comparator set because their favorable QSAR predictions were outside the practical descriptor-domain limits. Such compounds should be treated as structurally informative comparators unless additional docking, dynamic, or experimental data support their further prioritization.

### 3.10. Molecular Docking Studies

**Figure 3:**
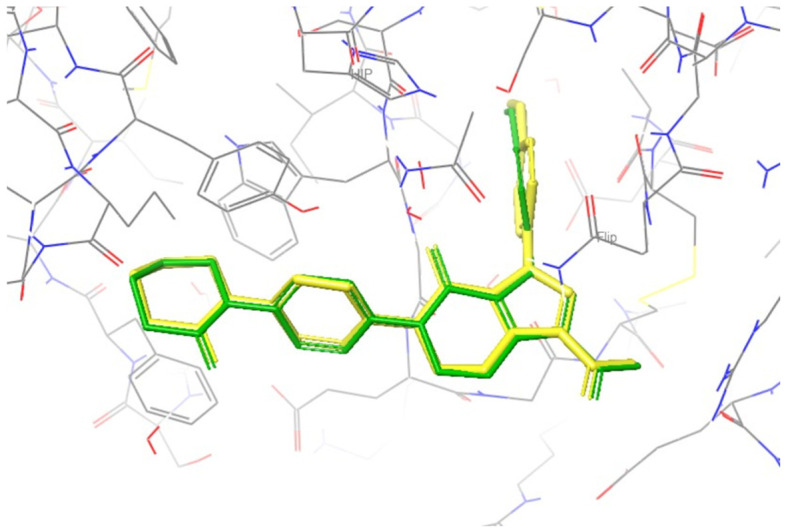
Validation of the molecular docking protocol by redocking of the co-crystallized apixaban molecule into the active site of human coagulation factor Xa (PDB ID: 2P16). The crystallographic ligand conformation is shown in green, whereas the redocked pose is shown in yellow. The excellent overlap between both conformations, reflected by a root-mean-square deviation (RMSD) value of 0.953 Å, confirms the reliability and accuracy of the applied docking procedure for subsequent studies of the investigated isosteviol derivatives.

The molecular docking protocol was initially validated through redocking of the co-crystallized inhibitor apixaban into the active site of human coagulation factor Xa (FXa, PDB ID: 2P16). The resulting pose reproduced the experimentally observed binding mode with a root-mean-square deviation (RMSD) of 0.953 Å (Figure 3), confirming the accuracy and reliability of the applied docking methodology. Furthermore, the redocked apixaban pose retained the characteristic interaction pattern reported for FXa inhibitors, supporting the suitability of the selected crystal structure for subsequent virtual screening of the investigated isosteviol derivatives.

The calculated docking scores (Table 9) revealed substantial differences in the predicted binding affinities of the studied compounds toward FXa. As expected, the reference inhibitor apixaban displayed the most favorable GlideScore (−11.362 kcal/mol). Among the investigated derivatives, ISV-M04 demonstrated the highest predicted affinity (−10.963 kcal/mol), closely approaching that of apixaban, whereas ISV-M19 also exhibited a highly favorable docking score (−10.041 kcal/mol). Intermediate scores were obtained for ISV-M06 (−9.795 kcal/mol), ISV-M17 (−9.715 kcal/mol), ISV-M16 (−9.310 kcal/mol), and ISV-M09 (−9.159 kcal/mol), while ISV-M20 (−8.858 kcal/mol) and ISV-M18 (−8.530 kcal/mol) showed comparatively weaker, yet still favorable, binding energies.

Analysis of the binding poses revealed that the highest-scoring compounds generally adopted binding modes resembling the overall interaction pattern observed for apixaban. In particular, ISV-M04 and ISV-M19 efficiently occupied both the hydrophobic S4 region and the deeper catalytic pocket containing Asp189, Ala190, Cys191, and Gln192. This dual-site occupation appears to be a major determinant of favorable ligand recognition within FXa. The aromatic substituents of both ligands penetrated deeply into the catalytic cavity, while the rigid diterpenoid scaffold remained positioned within the hydrophobic region formed by Tyr99, Thr98, and Lys96. Such an arrangement enabled extensive van der Waals interactions and optimal geometric complementarity with the enzyme active site. Notably, the binding mode of ISV-M04 exhibited remarkable similarity to that of apixaban, which likely explains its exceptionally favorable docking score.

ISV-M19 adopted a closely related orientation and exploited a largely overlapping set of residues. However, subtle differences in the aromatic fragment and its electronic properties resulted in slightly less favorable predicted binding. Nevertheless, the overall interaction network remained highly extensive, supporting the classification of ISV-M19 as one of the most promising compounds within the investigated series. The similarity between ISV-M04 and ISV-M19 suggests that efficient simultaneous occupation of both S1 and S4 regions may represent an advantageous structural feature for future optimization of isosteviol-based FXa inhibitors.

A different binding strategy was observed for ISV-M06 and ISV-M17. Rather than maximizing interactions within the Asp189-containing region, both ligands relied more heavily on aromatic and hydrophobic stabilization within the Tyr99–Trp215 region of the binding pocket. Particularly noteworthy was the participation of Trp215 in π-type interactions with the aromatic heterocyclic substituents. Such interactions are frequently observed among potent FXa inhibitors and are known to contribute significantly to ligand stabilization. The fluorinated aromatic fragment present in ISV-M17 appeared especially well accommodated within this hydrophobic environment, which likely explains its slightly more favorable docking score compared with ISV-M06. In addition, ISV-M17 retained stabilizing contacts involving Gly216 and Glu217, thereby combining favorable aromatic interactions with occupation of central regions of the catalytic cavity.

The binding mode of ISV-M09 differed substantially from those described above. In this case, stabilization was largely associated with a water-mediated interaction network involving Gly216 and Glu217. These residues also participate in interactions with apixaban, highlighting their importance within the FXa binding pocket. Although ISV-M09 displayed fewer interactions with the deepest portion of the catalytic cavity, the presence of water-bridged contacts and extensive hydrophobic interactions allowed productive accommodation within the active site. This observation emphasizes the potential role of solvent-mediated interactions in stabilizing ligand binding and suggests that docking scores alone may not fully capture the energetic contribution of such interactions.

ISV-M16 displayed another distinct binding pattern. While the ligand occupied the Asp189-containing region relatively effectively, it additionally formed a direct hydrogen bond involving Gln61. This interaction was not observed for most other derivatives and provided an additional anchoring point within the active site. Despite this favorable interaction, the overall docking score remained lower than those obtained for ISV-M04 and ISV-M19, indicating that optimal binding is determined by the collective balance of interactions throughout the entire pocket rather than by the presence of a single strong contact. The binding mode of ISV-M16 therefore highlights the complexity of ligand recognition by FXa and the importance of global rather than local complementarity.

The lowest-scoring compounds, ISV-M18 and ISV-M20, nevertheless exhibited productive binding modes and remained well accommodated within the catalytic pocket. Both ligands preferentially occupied the hydrophobic S4 region and established aromatic interactions with residues such as Tyr99, Phe174, and Trp215. However, their engagement with the deeper catalytic region was less extensive than that observed for the top-ranked derivatives. Consequently, the resulting interaction networks were comparatively limited, leading to less favorable docking scores. Interestingly, ISV-M20 had previously been identified as a promising candidate by QSAR analysis, suggesting that structural features associated with predicted biological activity do not necessarily translate directly into optimal static binding within the crystallographic receptor structure.

**Figure 4:**
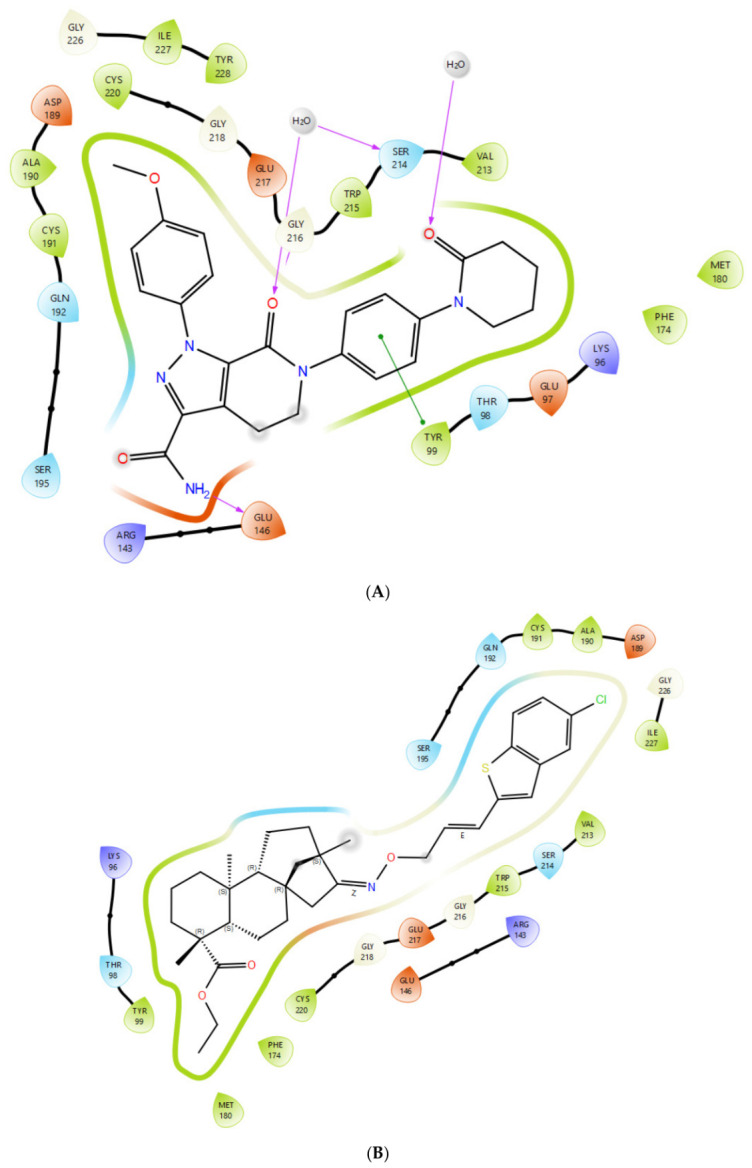

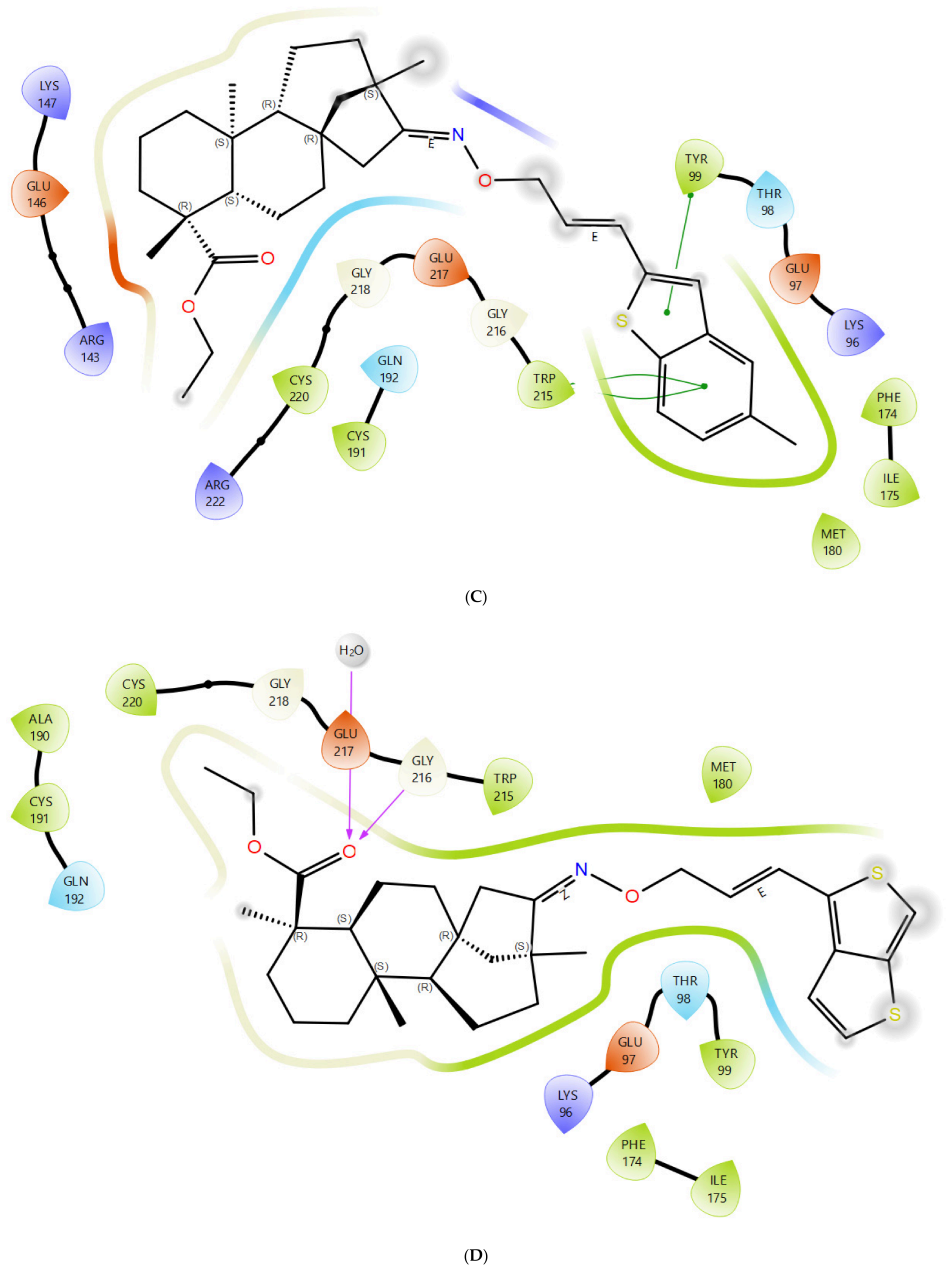

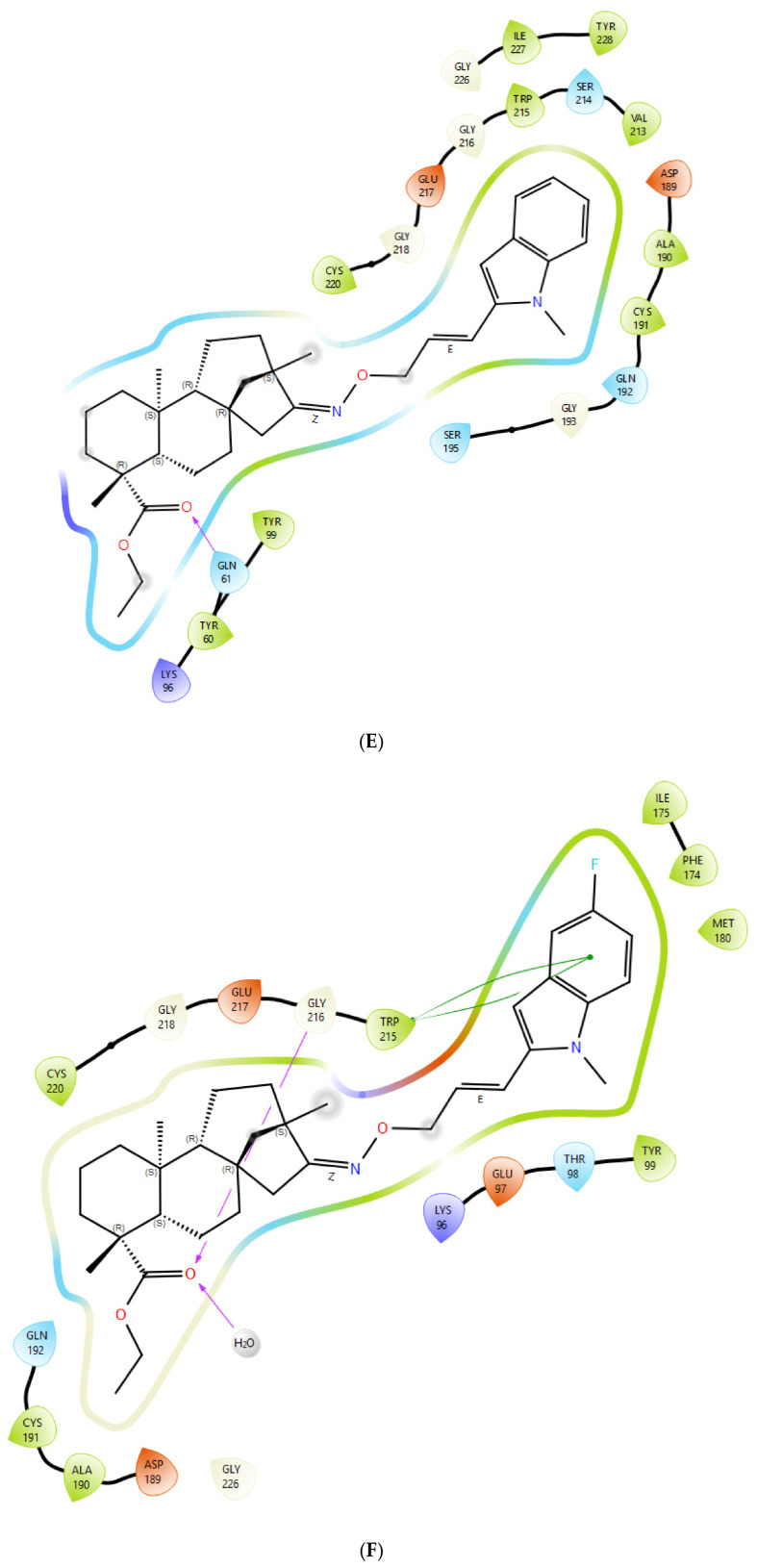

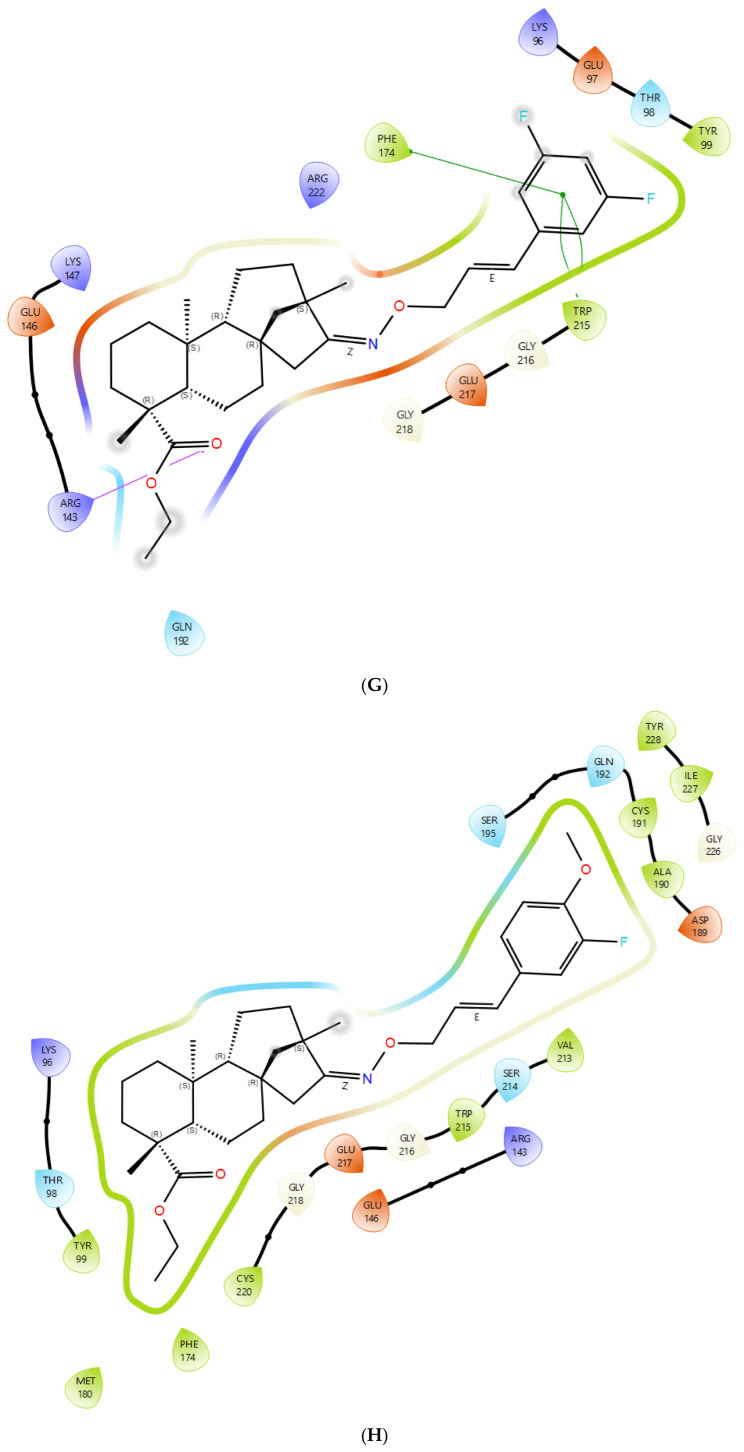

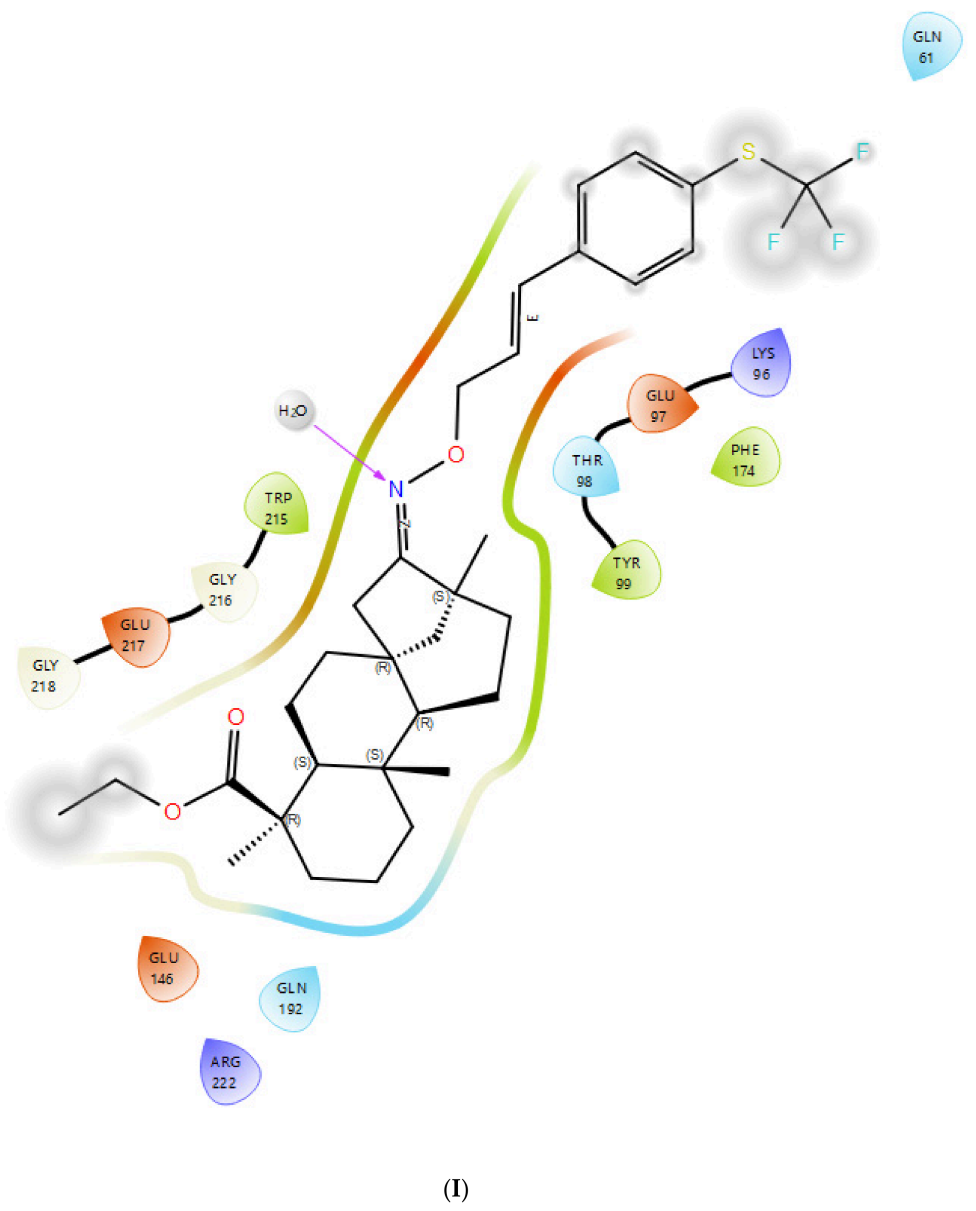
Two-dimensional protein–ligand interaction diagrams for the co-crystallized FXa inhibitor apixaban and the investigated ISV derivatives docked into the active site of factor Xa. Panels represent (**A**) Apixaban, (**B**) ISV-M04, (**C**) ISV-M06, (**D**) ISV-M09, (**E**) ISV-M16, (**F**) ISV-M17, (**G**) ISV-M18, (**H**) ISV-M19, and (**I**) ISV-M20. The diagrams illustrate the key intermolecular interactions responsible for ligand recognition and stabilization within the binding pocket, including hydrogen bonding, hydrophobic contacts, aromatic interactions, and electrostatic contacts. Only the top-scoring docking pose for each ligand is shown.

Overall, the docking results (Figure 4) indicate that efficient occupation of both the hydrophobic S4 pocket and the Asp189-containing catalytic region represents the most favorable binding strategy for the investigated isosteviol derivatives. Compounds ISV-M04 and ISV-M19 emerged as the strongest binders according to docking calculations, while ISV-M17 and ISV-M06 also displayed highly favorable interaction patterns. At the same time, the diversity of observed binding modes suggests that ligand recognition by FXa can be achieved through multiple interaction strategies involving different combinations of hydrophobic contacts, aromatic interactions, hydrogen bonds, and water-mediated stabilization. Because molecular docking provides only a static representation of protein–ligand recognition, all docked complexes were subsequently subjected to molecular dynamics simulations to evaluate the stability of the predicted binding modes under dynamic conditions and to further characterize the energetic determinants of ligand binding.

### 3.11. Molecular Dynamics Simulations and Binding Free Energy Analysis

To further evaluate the stability of the docked complexes and investigate the dynamic behavior of the investigated ligands within the FXa active site, initial 100 ns molecular dynamics simulations were performed for apixaban and all ISV derivatives. For the four key complexes selected for detailed receptor-based evaluation, namely FXa–apixaban, FXa–ISV-M19, FXa–ISV-M04, and FXa–ISV-M06, additional continuous 200 ns production simulations were then performed and used as the primary basis for the final quantitative stability analysis. Structural stability was assessed using protein and ligand root-mean-square deviation (RMSD) profiles, whereas the persistence of intermolecular interactions was analyzed using protein–ligand contact occupancy maps. In addition, binding affinities were estimated by MM/GBSA calculations (Table 9) based on the MD trajectories.

The protein backbone remained highly stable throughout all simulations, with RMSD values generally fluctuating between 1.0 and 1.6 Å, indicating that no major conformational changes occurred in FXa during the simulations (Figure S1). Such behavior demonstrates that the observed differences between ligands mainly originated from their intrinsic flexibility and interaction patterns (Figure S2) rather than from protein instability.

The reference inhibitor apixaban displayed an initially elevated ligand RMSD, followed by stabilization after approximately 35 ns. During the second half of the simulation, the ligand adopted a stable binding mode with RMSD values close to 1 Å, indicating a well-defined orientation inside the catalytic pocket. Persistent interactions with Tyr60, Tyr99, Phe94, Lys96, Phe174 and Trp215 were observed, which is consistent with the known binding mode of FXa inhibitors. Accordingly, apixaban exhibited the most favorable MM/GBSA binding free energy (−74.70 kcal/mol).

Among the investigated derivatives, ISV-M04 showed one of the most stable trajectories. The ligand RMSD fluctuated around 1.5–2.0 Å throughout the simulation without major conformational transitions. Protein–ligand contact analysis revealed that binding was dominated by Trp215 and Phe174, accompanied by interactions with Tyr99 and Glu97. The relatively balanced interaction network translated into a favorable MM/GBSA energy of −66.71 kcal/mol, representing the second-best binding affinity among the ISV derivatives.

Similarly, ISV-M06 exhibited excellent stability during the 100 ns simulation. Although a conformational adjustment was observed around 80 ns, the ligand rapidly converged to another stable orientation. Persistent interactions involved Tyr99, Trp215, Phe174, and Lys96, which collectively provided efficient stabilization within the S1 pocket. Consequently, ISV-M06 showed a favorable ΔG_bind value of −65.38 kcal/mol. The combination of low RMSD fluctuations and persistent interactions suggests that this derivative may represent one of the most promising compounds in the series.

ISV-M09 demonstrated moderate stability. The RMSD profile indicated a relatively stable trajectory with only a short-lived rearrangement near 50 ns. The interaction occupancy diagram revealed particularly strong contributions from Tyr99 and Trp215, whereas Phe174 also participated in ligand stabilization. Although these contacts were highly persistent, the overall interaction network was less diversified than for M04 and M06, resulting in a somewhat weaker binding free energy (−52.73 kcal/mol).

ISV-M16 displayed a more complex behavior characterized by several transient conformational changes. Ligand RMSD remained relatively high throughout the simulation and occasional rearrangements were evident. The interaction profile showed significant contributions from Val213 and Tyr99, accompanied by contacts with Gly216 and Ile175. Despite the presence of persistent interactions, the overall binding pattern appeared less organized, which was reflected by the relatively weak MM/GBSA energy of −55.61 kcal/mol.

ISV-M17 initially adopted a stable binding mode but underwent substantial conformational transitions after approximately 80 ns. Protein–ligand contact analysis revealed highly persistent interactions with Tyr99, Phe174 and Trp215, resembling the interaction pattern observed for potent FXa inhibitors. Nevertheless, the RMSD profile demonstrated that these contacts were associated with multiple binding orientations rather than with a single well-defined pose. As a consequence, the favorable contact occupancy was not accompanied by equally favorable binding energies, and the calculated ΔG_bind reached only −53.11 kcal/mol.

A similar phenomenon was observed for ISV-M18. This ligand displayed exceptionally high occupancies for Trp215 and Phe174, both exceeding 85%, as well as substantial interactions with Tyr99. However, the RMSD profile showed a marked conformational transition after approximately 70–75 ns, suggesting the formation of an alternative binding arrangement. Thus, despite apparently excellent contact persistence, the overall binding free energy remained relatively weak (−52.33 kcal/mol). These findings indicate that high interaction occupancies alone do not necessarily guarantee strong binding and that the spatial arrangement of the interactions is equally important.

Among all investigated derivatives, ISV-M19 exhibited the most balanced and coherent behavior. The RMSD profile showed excellent convergence and only minor fluctuations during the entire simulation. Unlike M17 and M18, no pronounced conformational transitions were observed. The protein–ligand contact analysis revealed a broad interaction network involving Trp215, Glu217, Ala190, Val213, Tyr99, Phe174, Gly216 and Tyr228. Instead of relying on one or two dominant contacts, M19 established numerous medium-strength interactions distributed over the binding pocket. Such a balanced interaction pattern resulted in a highly favorable MM/GBSA binding free energy of −68.23 kcal/mol, which was surpassed only by the reference inhibitor. The excellent agreement between RMSD stability, contact persistence and binding energy strongly suggests that M19 represents the most promising derivative in the investigated series.

In contrast, ISV-M20 exhibited the least favorable dynamic behavior. The ligand RMSD fluctuated considerably during the simulation and no clear plateau was reached, indicating continuous rearrangements within the binding pocket. Furthermore, the interaction profile differed substantially from those of the other ligands. The characteristic FXa residues Tyr99, Phe174, Trp215 and Gly216 contributed only marginally, whereas the dominant interactions involved Lys64 and Lys146. Such an interaction pattern suggests that M20 failed to exploit the canonical binding regions of factor Xa. Consequently, this compound exhibited the weakest MM/GBSA energy (−52.00 kcal/mol), confirming its inferior binding properties.

To address the stability of the key complexes over a longer continuous simulation window, 200 ns MD simulations were performed for FXa–apixaban, FXa–ISV-M19, FXa–ISV-M04, and FXa–ISV-M06. The extended trajectories showed that the protein backbone remained within an acceptable RMSD range and did not undergo major structural disruption during the simulations. The ligand RMSD and protein–ligand contact profiles further supported stable accommodation of the prioritized ISV derivatives within the FXa binding pocket. These extended simulations therefore strengthen, but do not fundamentally alter, the original receptor-based prioritization of ISV-M19, ISV-M04, and ISV-M06.

To provide a more quantitative comparison of complex stability, the RMSD profiles obtained from the 200 ns MD simulations were further analyzed by determining the approximate plateau time and calculating mean RMSD values with standard deviations over the equilibrated portion of each trajectory (Table 10). The RMSD plateau was defined as the time point after which both the protein backbone RMSD and ligand RMSD fluctuated around a stable average without sustained drift. Because ligand RMSD equilibration occurred later than protein backbone equilibration, a conservative common equilibrated interval of 150–200 ns was used for the final comparison of the four key complexes.

The FXa backbone RMSD remained low for all investigated complexes, with mean values of 1.36 ± 0.09 Å for FXa–apixaban, 1.30 ± 0.11 Å for FXa–ISV-M19, 1.40 ± 0.12 Å for FXa–ISV-M04, and 1.39 ± 0.16 Å for FXa–ISV-M06. These values indicate that the protein structure remained stable and did not undergo major conformational rearrangement during the extended simulations. The ligand RMSD values also supported stable ligand behavior during the equilibrated portion of the trajectories. FXa–ISV-M19 displayed the lowest ligand RMSD among the prioritized isosteviol derivatives, with a mean value of 1.55 ± 0.50 Å. FXa–ISV-M04 showed a ligand RMSD of 1.68 ± 0.96 Å, comparable to apixaban, whereas FXa–ISV-M06 showed a higher value of 2.47 ± 0.76 Å, indicating somewhat greater ligand flexibility. Overall, the quantitative RMSD analysis supports the conclusion that the prioritized ISV derivatives, particularly ISV-M19 and ISV-M04, remained stably accommodated in the FXa binding site during the 200 ns MD simulations.

To complement the RMSD-based stability assessment, additional MD descriptors were analyzed for the four key FXa–ligand complexes using the extended 200 ns trajectories. (Table 11) Residue-wise RMSF analysis of the principal binding-site residues Lys96, Tyr99, Phe174, Asp189, Gln192, Trp215, and Gly216 showed limited fluctuations, with mean Cα RMSF values of 0.79 Å for FXa–apixaban, 0.81 Å for FXa–ISV-M19, 0.71 Å for FXa–ISV-M04, and 0.71 Å for FXa–ISV-M06. These results indicate that the FXa binding pocket remained structurally stable in all analyzed complexes.

Protein radius of gyration was further used to assess the global compactness of FXa during the simulations. The mean protein Rg values calculated over the equilibrated 150–200 ns interval were 18.82 ± 0.06 Å for FXa–apixaban, 18.83 ± 0.06 Å for FXa–ISV-M19, 18.78 ± 0.09 Å for FXa–ISV-M04, and 18.77 ± 0.06 Å for FXa–ISV-M06. The very small variation in protein Rg indicates that no global destabilization or unfolding-like behavior occurred during the extended simulations.

Protein–ligand contact analysis showed that ligand stabilization was governed mainly by hydrophobic/aromatic contacts and, in some cases, water-mediated or hydrogen-bond interactions. ISV-M19 maintained persistent hydrophobic contacts with Phe174, Trp215, and Tyr99, together with water bridges involving Glu97, Glu217, and Ser173. ISV-M04 showed a strong hydrogen-bond interaction with Tyr99 and additional stabilization through Trp215 hydrophobic and π–π contacts. ISV-M06 was stabilized mainly by hydrophobic contacts with Trp215, Tyr60, Phe41, Phe174, and Tyr99, together with a π–cation interaction involving Arg143. No persistent ionic or metal-mediated contacts were observed.

Trajectory-derived ligand-property analysis showed mean ligand rGyr values of 5.49 ± 0.27 Å for apixaban, 5.29 ± 0.38 Å for ISV-M19, 5.57 ± 0.41 Å for ISV-M04, and 5.95 ± 0.29 Å for ISV-M06, indicating that the ligands retained compact bound conformations during the equilibrated portions of the extended trajectories. Together with the MM/GBSA results, these analyses strengthen the receptor-based prioritization of ISV-M19, ISV-M04, and ISV-M06.

An exploratory statistical analysis was performed for the main MD-derived descriptors obtained from the equilibrated 150–200 ns intervals of the extended trajectories (Table 12). Pairwise comparisons of 5 ns block-averaged backbone RMSD values showed no increase in protein structural deviation for any prioritized ISV complex relative to FXa–apixaban. FXa–ISV-M19 showed a slightly lower backbone RMSD than FXa–apixaban, whereas the differences for FXa–ISV-M04 and FXa–ISV-M06 were not statistically significant. Ligand RMSD comparisons showed no statistically significant difference for ISV-M19 or ISV-M04 relative to apixaban, while ISV-M06 displayed significantly higher ligand RMSD, indicating somewhat greater internal ligand flexibility. Importantly, neither protein radius of gyration nor residue-wise RMSF analysis indicated ligand-induced destabilization of the FXa structure or binding pocket. These statistical results support the conclusion that the prioritized ISV derivatives remain structurally compatible with the FXa active site during the extended MD simulations.

Overall, the molecular dynamics simulations, protein–ligand contact analysis, and MM/GBSA calculations consistently identified ISV-M19, ISV-M04 and ISV-M06 as the most promising derivatives. In particular, ISV-M19 combined excellent structural stability, a broad and balanced interaction network, and highly favorable binding energetics. The convergence of all three computational approaches strengthens the reliability of these findings and indicates that these compounds may represent attractive candidates for further experimental evaluation as potential factor Xa inhibitors.

### 3.12. In Silico ADMET and Toxicity Profiling

To complement the activity- and binding-oriented prioritization workflow, the eight compounds evaluated by docking, molecular dynamics, and MM/GBSA were subjected to preliminary SwissADME and pkCSM profiling (Table 13). All prioritized analogs were predicted to have high lipophilicity, poor aqueous solubility, low gastrointestinal absorption, and no blood–brain barrier permeation. These features are consistent with the bulky tetracyclic isosteviol scaffold and indicate that the designed compounds should be regarded as early lead-like analogs requiring further optimization rather than as directly drug-like candidates. At the same time, all eight compounds were free of PAINS alerts, satisfied the Veber filter, and showed no predicted AMES toxicity, hERG I inhibition, hepatotoxicity, or skin-sensitization liability.

Among the receptor-based top candidates, ISV-M04, ISV-M06, and ISV-M19 showed the cleanest pkCSM toxicity profiles, with no predicted AMES, hERG I, hERG II, hepatotoxicity, or skin-sensitization alerts. This finding supports the prioritization of these compounds, especially ISV-M19, which combined favorable docking, stable molecular-dynamics behavior, strong MM/GBSA binding free energy, and an acceptable preliminary toxicity screen. In contrast, ISV-M20, although ranked highest by the QSAR model, showed a less favorable developability profile because of very high lipophilicity, poor predicted solubility, P-gp substrate status, and a predicted hERG II liability. Thus, the ADMET/toxicity screen further supports the interpretation that ligand-based QSAR predictions should be integrated with receptor-based and developability-oriented filters before selecting compounds for synthesis and biological testing.

## 4. Discussion

The present study demonstrates that the combination of interpretable QSAR modeling with structure-based computational approaches constitutes a powerful strategy for the rational design of novel factor Xa inhibitors. While descriptor-based models are particularly useful for identifying global structure–activity relationships, molecular docking and molecular dynamics simulations provide complementary information regarding the stability and persistence of protein–ligand interactions within the receptor environment. Consequently, these approaches should be regarded as synergistic rather than redundant tools in computer-aided drug design.

The constrained second-order MARSplines model developed in this work exhibited excellent internal performance and satisfactory robustness despite being based on a relatively small congeneric dataset. Importantly, the use of a compact model involving only four active basis functions enabled straightforward interpretation of the structural factors governing biological activity. Such parsimonious models are particularly advantageous because they reduce the risk of overfitting and facilitate mechanistic understanding of structure–activity relationships. The descriptor combination identified in the present work indicates that both electronic and topological characteristics contribute significantly to FXa inhibitory potency.

Application of the model to the virtually designed series of twenty new isosteviol derivatives identified ISV-M20 as the most promising candidate within the applicability domain. However, subsequent structure-based investigations revealed that high descriptor-based activity predictions do not necessarily guarantee the most favorable dynamic interaction profile within the receptor environment. This observation highlights the importance of combining ligand-based and receptor-based computational approaches during lead optimization. Interestingly, the discrepancy between the QSAR predictions and the molecular dynamics results highlights the complementary nature of ligand-based and structure-based approaches. Whereas QSAR models capture statistical relationships derived from known compounds, molecular dynamics simulations provide insight into the temporal stability of ligand binding and may therefore reveal limitations associated with descriptor-based extrapolation.

A major strength of the present workflow is that the MARSplines QSAR model was used as an interpretable design and prioritization filter rather than as a stand-alone decision tool. The ligand-based model provided descriptor-level information on the structural requirements within the isosteviol oxime ether chemical space and enabled the design of a focused analog panel. Subsequent docking, molecular dynamics, MM/GBSA, and ADMET/toxicity filtering refined this initial ranking by evaluating binding-mode stability, persistence of key interactions, relative binding free energies, and preliminary developability. This sequential interpretation explains why ISV-M20, although favored by the descriptor-based model, was not selected as the final leading candidate, whereas ISV-M19, ISV-M04, and ISV-M06 emerged as more balanced compounds. Thus, the combined workflow generated both descriptor-level structure–activity information and receptor-level prioritization for future synthesis and biological evaluation. This interpretation is consistent with recent factor Xa inhibitor design studies integrating ligand-based, structure-based, and experimental/developability layers [38,39].

Docking studies demonstrated that all selected derivatives were capable of occupying the active site of factor Xa with favorable GlideScores. Moreover, the docking protocol was successfully validated by redocking of the co-crystallized ligand apixaban, yielding an RMSD value of 0.953 Å, which confirms the reliability of the adopted docking methodology. Although docking scores suggested generally favorable binding for all investigated compounds, molecular dynamics simulations provided a more realistic representation of ligand behavior under dynamic conditions.

Apixaban should therefore be interpreted as a crystallographic binding-site reference and docking-validation ligand rather than as a ligand-based similarity template for the isosteviol derivatives. The designed compounds are not close structural analogs of apixaban and were not included in the QSAR training set. Because experimental co-crystal structures are not available for the isosteviol-derived analogs, their assignment to the apixaban-defined active-site cleft should be regarded as a structure-based computational hypothesis supported by docking, protein–ligand interaction analysis, and MD simulations.

The RMSD profiles obtained during the 100 ns simulations indicated that the protein backbone remained relatively stable for all complexes, with Cα RMSD values generally fluctuating around 1.1–1.5 Å. This observation suggests that none of the investigated ligands induced substantial structural perturbations of factor Xa. However, differences became more pronounced when ligand RMSD values were considered. ISV-M04 displayed remarkably stable behavior throughout the simulation, with only minor fluctuations and no evidence of major conformational rearrangements. Similarly, ISV-M06 exhibited stable trajectories during most of the simulation time, although a transition to an alternative binding mode was observed after approximately 80 ns. In contrast, ISV-M20 showed considerably larger fluctuations and frequent changes in ligand orientation, suggesting a less favorable accommodation within the binding pocket despite its high predicted activity.

Protein–ligand contact analysis further emphasized the differences among individual derivatives. In agreement with the docking results, aromatic residues TYR99, PHE174, and TRP215 emerged as key contributors to ligand stabilization. Persistent hydrophobic interactions involving these residues were observed for most complexes and appear to represent important determinants of favorable binding. Among all compounds, ISV-M19 exhibited the most extensive and persistent interaction network. In addition to strong contacts with TRP215 and PHE174, interactions involving TYR99 and several neighboring residues remained highly populated throughout the simulation. Such behavior indicates efficient stabilization within the S1 and adjacent subsites of factor Xa.

ISV-M06 also demonstrated extensive interactions with TYR99 and TRP215, whereas ISV-M04 was characterized by particularly persistent contacts with TRP215 and PHE174. These findings are noteworthy because aromatic residues located within the FXa active site are known to play a critical role in ligand recognition and stabilization. The importance of these residues is further supported by the interaction pattern observed for apixaban, which maintained similar contacts during the simulation and thus served as an appropriate reference system. This residue-level interpretation is also consistent with recent direct FXa inhibitor discovery reports emphasizing the S1/S4 recognition environment [7,38,39].

Binding free energies obtained from MM/GBSA calculations were largely consistent with the dynamic behavior observed in the simulations. Apixaban exhibited the most favorable ΔG_bind_ value (−74.70 kcal/mol), which is in agreement with its established clinical efficacy. Among the designed derivatives, ISV-M19 showed the strongest predicted binding affinity (−68.23 kcal/mol), closely followed by ISV-M04 (−66.71 kcal/mol) and ISV-M06 (−65.38 kcal/mol). These compounds also demonstrated favorable RMSD profiles and persistent intermolecular contacts, indicating a high degree of consistency among the different computational approaches. In contrast, ISV-M17, ISV-M18, and ISV-M20 displayed less favorable binding free energies and more pronounced dynamic fluctuations, suggesting reduced overall stability of the corresponding complexes.

Taken together, the results indicate that ISV-M19 represents one of the most promising compounds within the investigated series. Its favorable GlideScore, highly negative MM/GBSA binding energy, stable molecular dynamics trajectory, and extensive interaction network suggest that this derivative may possess particularly advantageous binding characteristics. Nevertheless, ISV-M04 and ISV-M06 also exhibited highly favorable dynamic properties and should therefore be regarded as valuable candidates for further investigation.

The additional ADMET and toxicity assessment further supports this integrated prioritization. The prioritized derivatives are large and highly lipophilic molecules with poor predicted aqueous solubility and low predicted gastrointestinal absorption, indicating that they should be regarded as early-stage lead-like structures rather than immediately optimized oral drug candidates. However, the absence of PAINS alerts and the lack of predicted AMES toxicity, hERG I inhibition, hepatotoxicity, and skin-sensitization liability for ISV-M04, ISV-M06, and ISV-M19 support their selection for further optimization. The predicted synthetic-accessibility scores also indicate that chemical synthesis may be challenging but feasible within a focused medicinal-chemistry program, particularly because the designed analogs preserve the same isosteviol core and oxime ether linker and differ only in the terminal aromatic substituent. In contrast, ISV-M20 showed a less favorable developability balance, including very high lipophilicity, poor solubility, P-gp substrate status, and a predicted hERG II alert, which provides an additional explanation for why it should not be prioritized solely on the basis of the QSAR score. Similar early ADMET-aware filtering has been used previously for isosteviol-derived anticoagulant candidates and relies on the same SwissADME/pkCSM framework applied here [13,36,37].

Several limitations of the present study should be acknowledged. First, the QSAR model was constructed using a relatively small congeneric dataset, which inherently limits its applicability outside the investigated isosteviol-derived oxime ether chemical space. Second, only eight representative compounds were subjected to docking, molecular dynamics simulations, and MM/GBSA calculations; therefore, additional designed analogs may still display favorable binding behavior. Third, MM/GBSA calculations provide approximate estimates of relative binding free energies and do not fully account for entropic effects or all solvent-mediated contributions. Fourth, SwissADME and pkCSM predictions should be interpreted only as preliminary developability indicators because in silico ADMET models depend on the chemical space and endpoints represented in their training data. Finally, all conclusions remain computational and require experimental validation. Future work should therefore prioritize the synthesis of ISV-M19, ISV-M04, and ISV-M06, followed by enzymatic FXa inhibition assays, plasma anticoagulant activity testing, selectivity profiling against related coagulation proteases, and experimental solubility, metabolic-stability, permeability, hERG, and hepatotoxicity assays. The proposed validation path is consistent with recent studies in which factor Xa inhibitor candidates were advanced from in silico prioritization to synthesis, enzymatic inhibition, selectivity assessment, and preliminary safety/developability evaluation [38,39]. A further limitation is that binding free energies were estimated using MM/GBSA rather than more computationally demanding endpoint or alchemical free-energy approaches. Therefore, the reported ΔG_bind_ values should be interpreted as relative ranking indicators supporting ligand prioritization, rather than definitive quantitative measures of binding affinity.

Despite these limitations, the present results demonstrate that combining interpretable QSAR modeling with molecular docking, molecular dynamics simulations, and binding free energy calculations provides a coherent framework for the identification and prioritization of novel factor Xa inhibitors. Such integrated approaches are increasingly recognized as valuable tools in modern drug discovery because they enable both mechanistic interpretation and efficient lead optimization prior to synthesis and biological evaluation. Recent CADD studies likewise support the use of integrated computational and experimental prioritization schemes in early anticoagulant discovery [38,39].

## 5. Conclusions

In the present study, a compact and internally validated constrained second-order MARSplines model was developed for a congeneric series of isosteviol-derived oxime ether inhibitors of factor Xa. The model demonstrated strong calibration and cross-validated predictive performance and provided a rational basis for the design of twenty new analogs differing only in the terminal aromatic fragment. This design strategy preserved the common isosteviol scaffold and oxime ether linker while enabling focused exploration of benzothiophene, benzothiazole, benzofuran, N-methylindole, and substituted phenyl fragments.

The combined docking, molecular dynamics, and MM/GBSA analyses showed that ligand-based QSAR ranking and receptor-based prioritization were complementary rather than interchangeable. Although ISV-M20 emerged as the highest-ranked compound according to the QSAR model, ISV-M19, ISV-M04, and ISV-M06 showed the most favorable overall receptor-based behavior, including stable trajectories, persistent interactions with key FXa residues, and advantageous binding free energies. In particular, ISV-M19 combined favorable docking, molecular dynamics, and MM/GBSA results, making it the strongest computational candidate within the investigated subset.

The preliminary ADMET/toxicity screen further strengthened this prioritization by showing that ISV-M19, ISV-M04, and ISV-M06 lacked the main pkCSM toxicity alerts observed for some other designed analogs, whereas ISV-M20 showed a less favorable developability profile despite its high QSAR-predicted potency. Overall, these findings support an integrated QSAR-, receptor-, and ADMET-aware strategy for prioritizing novel isosteviol-derived FXa inhibitor candidates. Future work should focus on the synthesis of ISV-M19, ISV-M04, and ISV-M06, followed by enzymatic FXa inhibition testing, plasma anticoagulant activity assays, selectivity studies against related coagulation proteases, and experimental ADMET/toxicity evaluation.

## Figures and Tables

**Table 1:** General scaffold and experimental FXa inhibitory activity of the isosteviol-derived training compounds used for QSAR model development.

General Scaffold of the 6ra-6rt Series 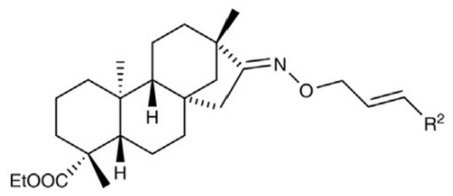				
Internal Code	Original Code	R2 Substituent	Ki_FXa [µM]	pKi_FXa
i_a	6ra	phenyl	10.697 ± 1.003	4.971
i_b	6rb	4-F-phenyl	6.169 ± 0.579	5.210
i_c	6rc	3-CF3-phenyl	4.861 ± 0.427	5.313
i_d	6rd	4-OCF3-phenyl	1.974 ± 0.127	5.705
i_e	6re	4-CN-phenyl	13.342 ± 1.210	4.875
i_f	6rf	4-NO2-phenyl	2.636 ± 0.256	5.579
i_g	6rg	3,4,5-F3-phenyl	1.980 ± 0.143	5.703
i_h	6rh	4-acetylphenyl	1.643 ± 0.153	5.784
i_i	6ri	4-(5-oxazole)phenyl	1.931 ± 0.162	5.714
i_j	6rj	4-(morpholinomethyl)phenyl	2.492 ± 0.210	5.603
i_k	6rk	bicyclic heteroaryl (X = NH)	4.674 ± 0.431	5.330
i_l	6rl	bicyclic heteroaryl (X = O)	10.276 ± 0.973	4.988
i_m	6rm	bicyclic heteroaryl (X = S)	0.211 ± 0.020	6.676
i_n	6rn	phthalide-type aryl	20.671 ± 2.312	4.685
i_o	6ro	dibenzofuran-type aryl (X = O)	20.984 ± 2.026	4.678
i_p	6rp	carbazole-type aryl (X = NH)	26.433 ± 3.169	4.578
i_q	6rq	indole-2-carboxylate-type aryl	1.913 ± 0.164	5.718
i_r	6rr	naphthyl	12.136 ± 1.353	4.916
i_s	6rs	quinolyl	4.177 ± 0.381	5.379
i_t	6rt	quinazolinyl	3.603 ± 0.342	5.443

Note: Ki values are expressed as mean ± SD and were taken from Chen et al. [10]. pKi_FXa values were calculated from the mean Ki_FXa values according to Equation (1). The general scaffold is shown to clarify the common isosteviol-derived oxime ether framework and the variable R2 position. Rivaroxaban was reported in the original study as a reference inhibitor but was not included in QSAR model development.

**Table 2:** Comparison of candidate QSAR models developed for FXa inhibitory activity prediction.

Model	Terms/ Description	No. Terms	R^2^	Adj. R^2^	Q^2^_LOO	RMSECV_LOO	MAE_LOO	Decision
MLR	MATS8i + Mor31p + R3u	3	0.799	0.761	0.652	0.289	0.214	Baseline
MLR	Mor31p + Mor31m + RGyr + R6p+	4	0.797	0.742	0.681	0.277	0.230	Baseline
Additive MARSplines	R6p+, C-025, ATSC7e, Mor31p; no interactions	4	0.905	0.880	0.854	0.187	0.148	Reference nonlinear model
Second-order MARSplines	R6p+, C-025, ATSC7e, Mor31p; pairwise interactions	4	0.929	0.910	0.865	0.180	0.139	Final model

**Table 3:** Basis functions included in the final constrained second-order MARSplines model.

Basis Function	Variables	Basis Function Definition	Coefficient
BF1	R6p+, Mor31p	h(R6p+ − 0.008) × h(Mor31p − 0.871)	+678.580929
BF2	C-025, Mor31p	h(C-025 − 1) × h(1.136 − Mor31p)	−1.648818
BF3	ATSC7e, Mor31p	h(0.496 − ATSC7e) × h(1.136 − Mor31p)	−100.735200
BF4	R6p+, ATSC7e	h(0.008 − R6p+) × h(0.547 − ATSC7e)	−8032.776775

**Table 4:** Statistical and validation parameters of the final MARSplines model.

Parameter	Value	Parameter	Value
Number of compounds	20	RMSEC	0.1308
Active descriptors	4	MAE calibration	0.0977
Basis functions	4	Q^2^_LOO	0.8646
Interaction degree	2	PRESS	0.6487
R^2^	0.9286	RMSECV_LOO	0.1801
Adjusted R^2^	0.9095	MAE_LOO	0.1388
Pearson r	0.9636	Repeated 5-fold Q^2^ mean	0.8363
CCC	0.9630	Repeated 5-fold Q^2^ SD	0.0520
Max VIF	1.1467	Repeated 5-fold RMSE mean	0.1958
Max |r| between basis functions	0.2602	Repeated 5-fold MAE mean	0.1491
F statistic	48.75	GCV	0.0304
*p* value	2.02 × 10^−8^	AICc	−67.08
BIC	−66.38		

**Table 5:** Y-randomization results for the final MARSplines model.

Metric	Value
Number of permutations	1000
Randomized models with R^2^ ≥ observed	0
Randomized models with Q^2^_LOO ≥ observed	0
Observed R^2^	0.9286
Maximum randomized R^2^	0.7582
Mean randomized R^2^	0.2206
Observed Q^2^_LOO	0.8646
Maximum randomized Q^2^_LOO	0.5731
Mean randomized Q^2^_LOO	−0.7334
95th percentile randomized Q^2^_LOO	−0.0006

**Table 6:** Descriptor ranges defining the practical applicability domain of the final model.

Descriptor	Minimum	Maximum	Mean	SD
ATSC7e	0.467	0.935	0.5389	0.1030
C-025	0.000	3.000	1.8000	0.8335
Mor31p	0.866	1.179	1.0131	0.0884
R6p+	0.007	0.013	0.0086	0.0016

**Table 7:** Leverage-based applicability domain summary.

Parameter	Value
Warning leverage threshold h*	0.75
Compounds above h*	i_a, i_m
Compounds with |standardized residual| > 3	none
Interpretation	i_a and i_m are influential compounds, but neither is a response outlier

**Table 8:** Model-guided series of twenty newly designed isosteviol-derived oxime ether analogs with predicted FXa inhibitory activity calculated using the final constrained second-order MARSplines model.

Code	Proposed R2 Substituent	Design Category	Priority	Predicted pKi_FXa (Ki_FXa [µM])
ISV-M01	5-F-benzo[b]thiophen-2-yl	Benzothiophene; conservative F analog	High	5.29 (5.17)
ISV-M02	6-F-benzo[b]thiophen-2-yl	Benzothiophene; positional F isomer	High	5.21 (6.21)
ISV-M03	5-Cl-benzo[b]thiophen-2-yl	Benzothiophene; polarizable Cl analog	High	5.53 (2.98) *
ISV-M04	6-Cl-benzo[b]thiophen-2-yl	Benzothiophene; positional Cl isomer	High	5.80 (1.59) *
ISV-M05	5-Me-benzo[b]thiophen-2-yl	Benzothiophene; small hydrophobic analog	High	3.98 (104.07) *
ISV-M06	6-Me-benzo[b]thiophen-2-yl	Benzothiophene; positional methyl isomer	High	5.56 (2.77) *
ISV-M07	5-OMe-benzo[b]thiophen-2-yl	Benzothiophene; electron-donating OMe	Medium/high	4.50 (31.67)
ISV-M08	benzo[b]thiophen-3-yl	Benzothiophene; alternative attachment point	High	4.89 (12.78)
ISV-M09	thieno[2,3-b]thiophen-5-yl	Sulfur-rich bicyclic heteroaryl	High	5.77 (1.70) *
ISV-M10	benzo[d]thiazol-2-yl	Benzothiazole bioisostere	High	3.98 (104.76) *
ISV-M11	5-F-benzo[d]thiazol-2-yl	Fluorinated benzothiazole	High	4.86 (13.96)
ISV-M12	5-Me-benzo[d]thiazol-2-yl	Methylated benzothiazole	Medium/high	5.42 (3.78) *
ISV-M13	5-F-benzofuran-2-yl	Fluorinated benzofuran comparator	Medium/high	5.11 (7.84)
ISV-M14	5-Me-benzofuran-2-yl	Methylated benzofuran comparator	Medium	4.58 (26.02) *
ISV-M15	5-OMe-benzofuran-2-yl	Methoxy benzofuran comparator	Medium	4.50 (31.97)
ISV-M16	1-Me-1H-indol-2-yl	N-methylindole heteroaryl analog	Medium/high	5.62 (2.38)
ISV-M17	5-F-1-Me-1H-indol-2-yl	Fluorinated N-methylindole	Medium/high	5.68 (2.11)
ISV-M18	3,5-difluorophenyl	Simple difluorophenyl comparator	Medium	5.63 (2.32)
ISV-M19	3-F-4-OMe-phenyl	Mixed F/OMe phenyl comparator	Medium	5.56 (2.77)
ISV-M20	4-SCF3-phenyl	Polarizable SCF3 phenyl	Medium	6.09 (0.81)

Note: Values are presented as pKi_FXa (Ki_FXa [µM]). Asterisks indicate predictions for which at least one of the four final-model descriptors fell outside the training-set descriptor range; these values should be interpreted as extrapolative.

**Table 9:** Glide XP docking scores and MM/GBSA binding free energies calculated for the investigated isosteviol derivatives and the reference inhibitor apixaban in complex with human coagulation factor Xa (FXa, PDB ID: 2P16). For FXa–apixaban, FXa–ISV-M19, FXa–ISV-M04, and FXa–ISV-M06, MM/GBSA values were calculated from frames extracted from the final 20 ns of the extended 200 ns trajectories. For the remaining ligand–FXa complexes, MM/GBSA values were calculated from frames extracted from the final 20 ns of the corresponding 100 ns trajectories. More negative GlideScore and MM/GBSA values indicate more favorable predicted ligand binding. MM/GBSA values were calculated from molecular dynamics trajectories using the Prime MM/GBSA method.

Compound	GlideScore (kcal/mol)	MM/GBSA ΔG_bind_ ± SD (kcal/mol)	Protein–Ligand Interactions
Apixaban	−11.36	−75.11 ± 2.07	H-bonds with Glu146 and Gly216, aromatic interactions with Tyr99
ISV-M04	−10.96	−67.01 ± 3.11	
ISV-M19	−10.04	−69.42 ± 4.21	
ISV-M06	−9.79	−64.83 ± 2.13	
ISV-M17	−9.71	−53.11 ± 4.38	H-bond with Gly216, aromatic interactions with Trp215
ISV-M16	−9.30	−55.61 ± 3.97	H-bond with Gln61
ISV-M09	−9.15	−52.73 ± 4.63	H-bond with Gly216
ISV-M20	−8.85	−52.00 ± 5.72	
ISV-M18	−8.52	−52.33 ± 4.97	Aromatic interactions with Phe174 and Trp215

**Table 10:** Quantitative RMSD analysis of the FXa–ligand complexes during the equilibrated portions of the 200 ns MD trajectories. The plateau time was estimated from the RMSD traces as the point after which the protein backbone and ligand RMSD fluctuated around a stable average without sustained drift. Mean values and standard deviations were calculated over the equilibrated portion of each trajectory.

Complex	Approx. RMSD Plateau	Equilibrated Interval	Backbone RMSD [Å]	Ligand RMSD, Lig_wrt_Ligand [Å]
FXa–apixaban	~150 ns	150–200 ns	1.36 ± 0.09	1.77 ± 0.72
FXa–ISV-M19	~150 ns	150–200 ns	1.30 ± 0.11	1.55 ± 0.50
FXa–ISV-M04	~150 ns	150–200 ns	1.40 ± 0.12	1.68 ± 0.96
FXa–ISV-M06	~150 ns	150–200 ns	1.39 ± 0.16	2.47 ± 0.76

**Table 11:** Additional MD descriptors for the key FXa–ligand complexes calculated from the extended 200 ns trajectories. Mean values and standard deviations for protein Rg and ligand rGyr were calculated over the equilibrated 150–200 ns interval.

Complex	Mean Key-Site Cα RMSF [Å]	Protein Rg [Å]	Ligand rGyr [Å]	Dominant Stabilizing Contacts
FXa–apixaban	0.79	18.82 ± 0.06	5.49 ± 0.27	Trp215, Tyr99, Phe174; water bridge with Gly216
FXa–ISV-M19	0.81	18.83 ± 0.06	5.29 ± 0.38	Phe174, Trp215, Tyr99; water bridges with Glu97, Glu217, Ser173
FXa–ISV-M04	0.71	18.78 ± 0.09	5.57 ± 0.41	Tyr99 H-bond; Trp215 hydrophobic/π–π contacts; Val213 hydrophobic contact
FXa–ISV-M06	0.71	18.77 ± 0.06	5.95 ± 0.29	Trp215, Tyr60, Phe41, Phe174, Tyr99; π–cation interaction with Arg143

**Table 12:** Exploratory statistical comparison of selected MD-derived descriptors for the equilibrated 150–200 ns portions of the 200 ns trajectories.

Metric	Comparison	*p*-Value	Interpretation
Backbone RMSD	Apixaban vs. ISV-M19	0.039	slightly lower for ISV-M19; no destabilization
Backbone RMSD	Apixaban vs. ISV-M04	0.142	no significant difference
Backbone RMSD	Apixaban vs. ISV-M06	0.642	no significant difference
Ligand RMSD	Apixaban vs. ISV-M19	0.245	no significant difference
Ligand RMSD	Apixaban vs. ISV-M04	0.805	no significant difference
Ligand RMSD	Apixaban vs. ISV-M06	0.024	higher flexibility for ISV-M06
Protein Rg	Apixaban vs. ISV-M19	0.143	no significant difference
Protein Rg	Apixaban vs. ISV-M04	0.174	no significant difference
Protein Rg	Apixaban vs. ISV-M06	0.003	slightly lower Rg for ISV-M06; no destabilization
Key-site Cα RMSF	Apixaban vs. ISV-M19	0.445	no significant difference
Key-site Cα RMSF	Apixaban vs. ISV-M04	0.115	no significant increase
Key-site Cα RMSF	Apixaban vs. ISV-M06	0.035	slightly lower RMSF; no pocket destabilization

**Table 13:** SwissADME and pkCSM summary for the eight prioritized ISV derivatives subjected to docking, molecular dynamics, and MM/GBSA calculations.

Compound	SwissADME Profile	Drug-Likeness/Medicinal Chemistry Alerts	pkCSM Toxicity Profile	ADMET-Based Interpretation
ISV-M04	MW 568.21; cLogP 8.74; ESOL LogS −9.02 (Poorly soluble); GI Low; BBB No; P-gp Yes	Lipinski No (2); Veber Yes; bioavailability score 0.17; PAINS 0; Brenk 2; SA 7.00	No AMES, hERG I, hepatotoxicity or skin-sensitization alerts	Favorable toxicity screen among prioritized compounds; main liabilities are high lipophilicity and poor solubility.
ISV-M06	MW 547.79; cLogP 8.49; ESOL LogS −8.73 (Poorly soluble); GI Low; BBB No; P-gp No	Lipinski No (2); Veber Yes; bioavailability score 0.17; PAINS 0; Brenk 2; SA 7.03	No AMES, hERG I, hepatotoxicity or skin-sensitization alerts	Favorable toxicity screen among prioritized compounds; main liabilities are high lipophilicity and poor solubility.
ISV-M09	MW 539.79; cLogP 8.24; ESOL LogS −8.29 (Poorly soluble); GI Low; BBB No; P-gp No	Lipinski No (2); Veber Yes; bioavailability score 0.17; PAINS 0; Brenk 2; SA 6.99	Alert(s): hERG II	Requires caution because of a predicted hERG II liability despite absence of AMES/hepatotoxicity alerts.
ISV-M16	MW 530.74; cLogP 7.03; ESOL LogS −7.80 (Poorly soluble); GI Low; BBB No; P-gp No	Lipinski No (2); Veber Yes; bioavailability score 0.17; PAINS 0; Brenk 2; SA 6.79	Alert(s): hERG II	Requires caution because of a predicted hERG II liability despite absence of AMES/hepatotoxicity alerts.
ISV-M17	MW 548.73; cLogP 7.29; ESOL LogS −7.97 (Poorly soluble); GI Low; BBB No; P-gp No	Lipinski No (2); Veber Yes; bioavailability score 0.17; PAINS 0; Brenk 2; SA 6.82	Alert(s): hERG II	Requires caution because of a predicted hERG II liability despite absence of AMES/hepatotoxicity alerts.
ISV-M18	MW 513.66; cLogP 7.77; ESOL LogS −7.70 (Poorly soluble); GI Low; BBB No; P-gp Yes	Lipinski No (2); Veber Yes; bioavailability score 0.17; PAINS 0; Brenk 2; SA 6.56	Alert(s): hERG II	Requires caution because of a predicted hERG II liability despite absence of AMES/hepatotoxicity alerts.
ISV-M19	MW 525.69; cLogP 7.33; ESOL LogS −7.62 (Poorly soluble); GI Low; BBB No; P-gp Yes	Lipinski No (2); Veber Yes; bioavailability score 0.17; PAINS 0; Brenk 2; SA 6.62	No AMES, hERG I, hepatotoxicity or skin-sensitization alerts	Favorable toxicity screen among prioritized compounds; main liabilities are high lipophilicity and poor solubility.
ISV-M20	MW 577.74; cLogP 8.68; ESOL LogS −8.91 (Poorly soluble); GI Low; BBB No; P-gp Yes	Lipinski No (2); Veber Yes; bioavailability score 0.17; PAINS 0; Brenk 2; SA 6.75	Alert(s): hERG II	Less favorable ADMET balance: highest lipophilicity/poor solubility, P-gp substrate status, and hERG II alert.

Note: cLogP denotes the SwissADME consensus Log Po/w. ESOL LogS is expressed in mol/L. SA denotes the SwissADME synthetic-accessibility score. Full SwissADME and pkCSM outputs for all twenty designed analogs are provided in the Supplementary Materials.

## Data Availability

The original contributions presented in this study are included in the article. Further inquiries can be directed to the corresponding author.

## Supplementary Materials

The following supporting information can be downloaded at: https://www.mdpi.com/article/10.3390/biology15141149/s1, Figure S1. Root-mean-square deviation (RMSD) of the protein backbone (blue) and ligands (magenta) as a function of simulation time for the co-crystallized FXa inhibitor apixaban and the investigated ISV derivatives, including replicate trajectories for the key ISV-M04, ISV-M06, and ISV-M19 complexes. Panels represent (A) Apixaban, (B–D) ISV-M04 replicate runs 1–3, (E–G) ISV-M06 replicate runs 1–3, (H) ISV-M09, (I) ISV-M16. The left y-axis corresponds to protein RMSD (Å), while the right y-axis corresponds to ligand RMSD (Å); Figure S2. Protein–ligand contact analysis for the co-crystallized FXa inhibitor apixaban and the investigated ISV derivatives obtained from molecular dynamics simulations. Panels represent (A) Apixaban, (B) ISV-M04, (C) ISV-M06, (D) ISV-M09, (E) ISV-M16, (F) ISV-M17, (G) ISV-M18, (H) ISV-M19. The interaction fraction indicates the proportion of simulation time during which a given amino acid residue remained in contact with the ligand. Colored segments denote different interaction types, including hydrogen bonds, hydrophobic interactions, water bridges, and ionic contacts. The figure supports the interpretation of key residues discussed in the manuscript, including Tyr99, Phe174, Trp215, Gly216, and Asp189. Persistent interactions are indicative of residues involved in ligand stabilization within the active site of factor Xa; Figure S3. Root-mean-square deviation (RMSD) of the protein backbone (blue) and ligands (magenta) as a function of simulation time for the co-crystallized FXa inhibitor apixaban and the investigated ISV derivatives, elongated production runs for the key Apixaban, ISV-M04, ISV-M06, and ISV-M19 complexes. Panels represent (A) Apixaban, (B) ISV-M04, (C) ISV-M06, (D) ISV-M19. The left y-axis corresponds to protein RMSD (Å), while the right y-axis corresponds to ligand RMSD (Å); Table S1. SwissADME physicochemical, drug-likeness, and medicinal-chemistry summary for ISV-M01-ISV-M20; Table S2. pkCSM toxicity prediction summary for ISV-M01-ISV-M20. Additional model diagnostics, descriptor matrices, applicability-domain outputs, and Gaussian input files are available from the corresponding author upon reasonable request.

## Abbreviations

The following abbreviations are used in this manuscript:

AD	applicability domain
CCC	concordance correlation coefficient
DFT	density functional theory
FXa	factor Xa
LOO	leave-one-out
MAE	mean absolute error
MARS	multivariate adaptive regression splines
MD	molecular dynamics
MLR	multiple linear regression
MM/GBSA	molecular mechanics generalized Born surface area
OPLS4	optimized potentials for liquid simulations 4
PRESS	predicted residual error sum of squares
QSAR	quantitative structure–activity relationship
RMSD	root mean square deviation
RMSE	root mean squared error
RMSEC	root mean squared error of calibration
RMSECV	root mean squared error of cross-validation
SAR	structure–activity relationship
SD	standard deviation
VIF	variance inflation factor
VSGB	variable dielectric generalized Born model
XP	extra precision
