# Supplementary material for: Rational Design of Novel Isosteviol-Derived Factor Xa Inhibitors Using Integrated QSAR, Molecular Docking, Molecular Dynamics, and MM/GBSA Analyses
Source: Biology (Basel). 2026 Jul 14;15(14):1149. doi: 10.3390/biology15141149 (PMC13405604; doi:10.3390/biology15141149)
Supplement: Supplementary file 1 [file biology-15-01149-s001.zip › biology-4414270-supplementary.pdf]

Supplementary Information

Rational Design of Novel Isosteviol-Derived Factor Xa Inhibitors Using Integrated QSAR, Molecular Docking, Molecular Dynamics, and MM/GBSA Analyses

Paweł Gordon [1], Łukasz Szeleszczuk [2], Małgorzata Lasota [3], Dariusz Maciej Pisklak [2,*] and Marcin Gackowski [4]

1 University of Health Sciences in Bydgoszcz, Jagiellońska 4 Str., 85-067 Bydgoszcz, Poland; p.gordon@wsnoz.edu.pl
2 Department of Organic and Physical Chemistry, Medical University of Warsaw, 1 Banacha Str., 02-097 Warsaw, Poland; lukasz.szeleszczuk@wum.edu.pl
3 Center for Biomedicine and Interdisciplinary Sciences, Faculty of Medicine, Jagiellonian University Medical College, 16 Grzegórzecka Street, 31-531 Krakow, Poland; malgorzata.lasota@uj.edu.pl
4 Department of Toxicology and Bromatology, Faculty of Pharmacy, L. Rydygier Collegium Medicum in Bydgoszcz, Nicolaus Copernicus University in Torun, A. Jurasza 2 Street, 85-089 Bydgoszcz, Poland; marcin.gackowski@cm.umk.pl
* Correspondence: dpisklak@wum.edu.pl

A

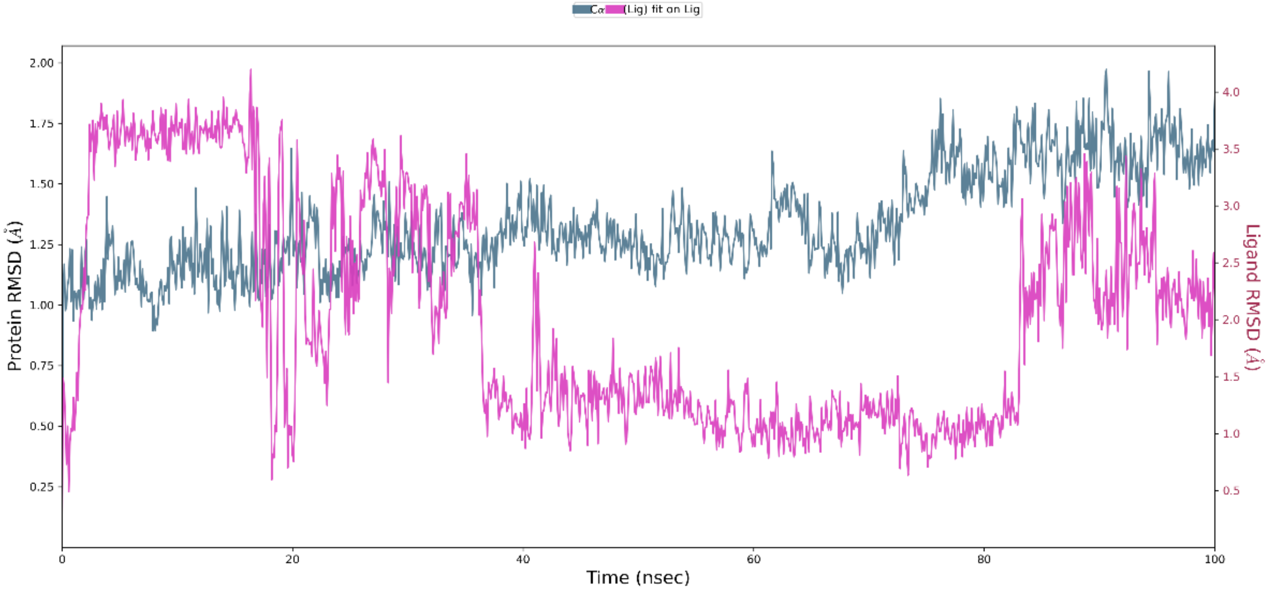

B

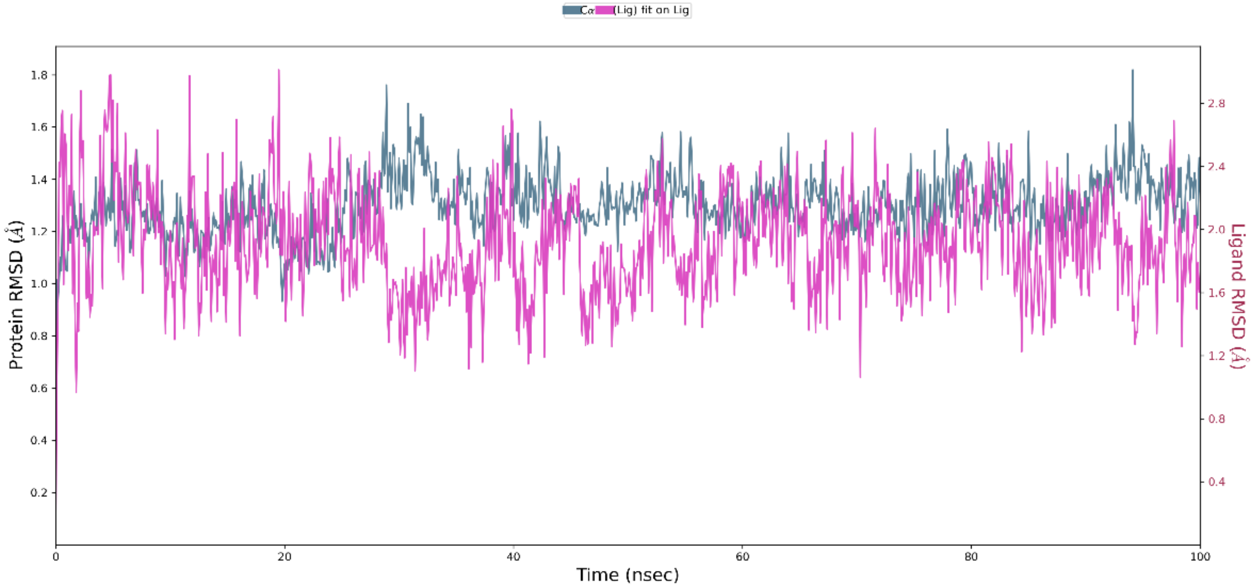

Cα (Lig) fit on Lig
Protein RMSD (Å)
Ligand RMSD (Å)
Time (nsec)

C

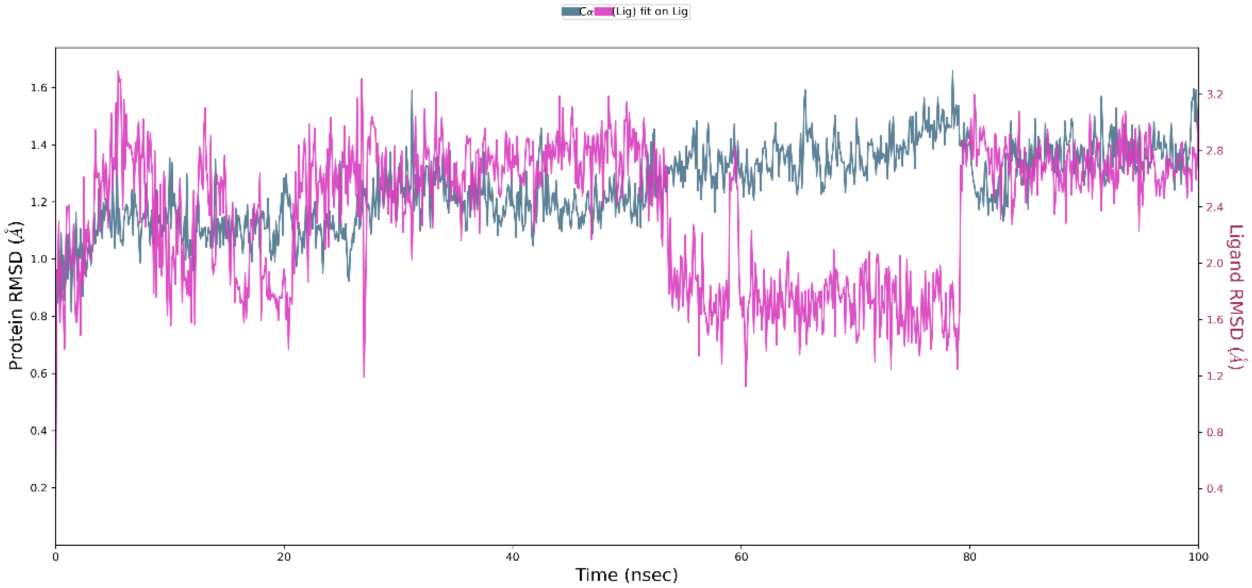

Cα (Lig) fit on Lig
Protein RMSD (Å)
Ligand RMSD (Å)
Time (nsec)

D

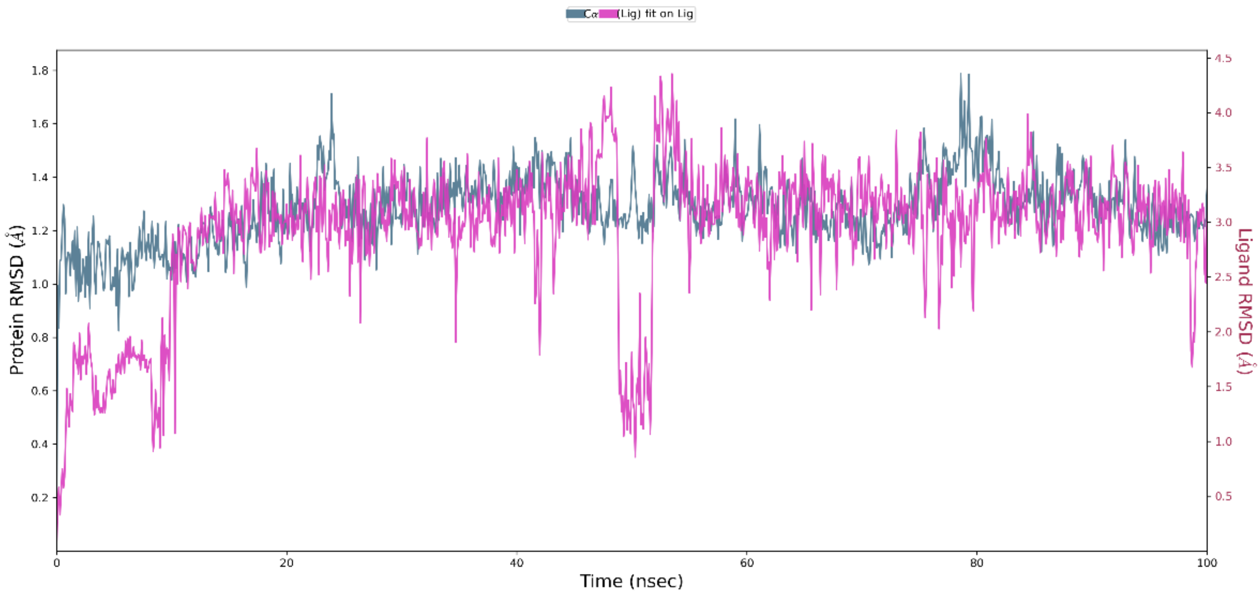

Cα
(Lig) fit on Lig
Protein RMSD (Å)
Ligand RMSD (Å)
Time (nsec)

E

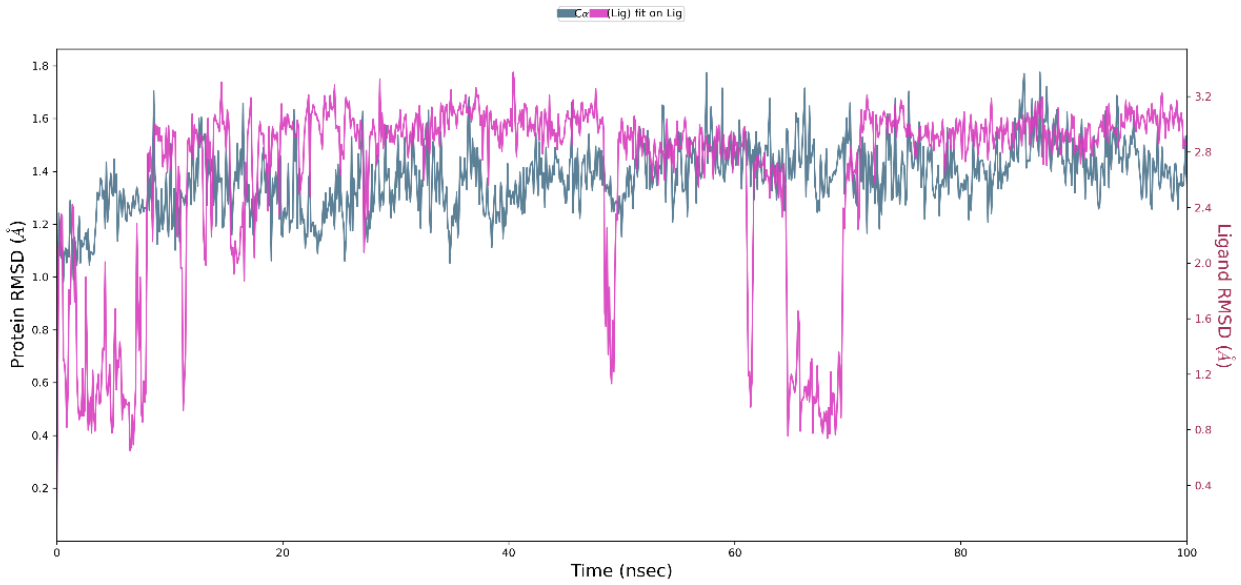

Cα
(Lig) fit on Lig
Protein RMSD (Å)
Ligand RMSD (Å)
Time (nsec)

F

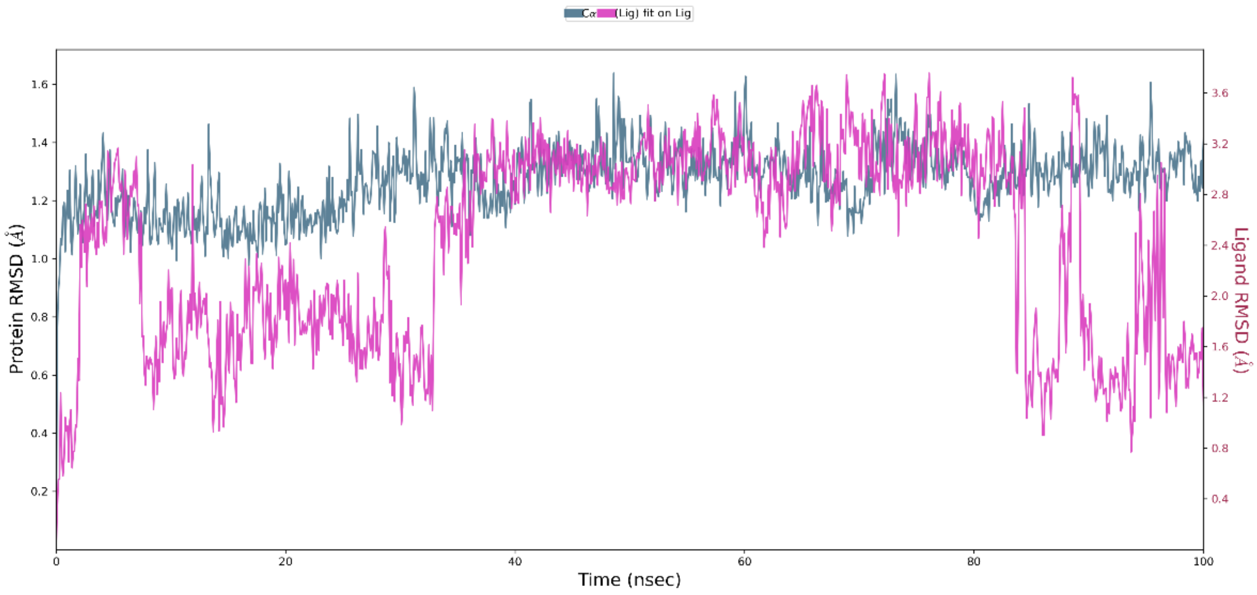

Cα (Lig) fit on Lig
Protein RMSD (Å)
Ligand RMSD (Å)
Time (nsec)

G

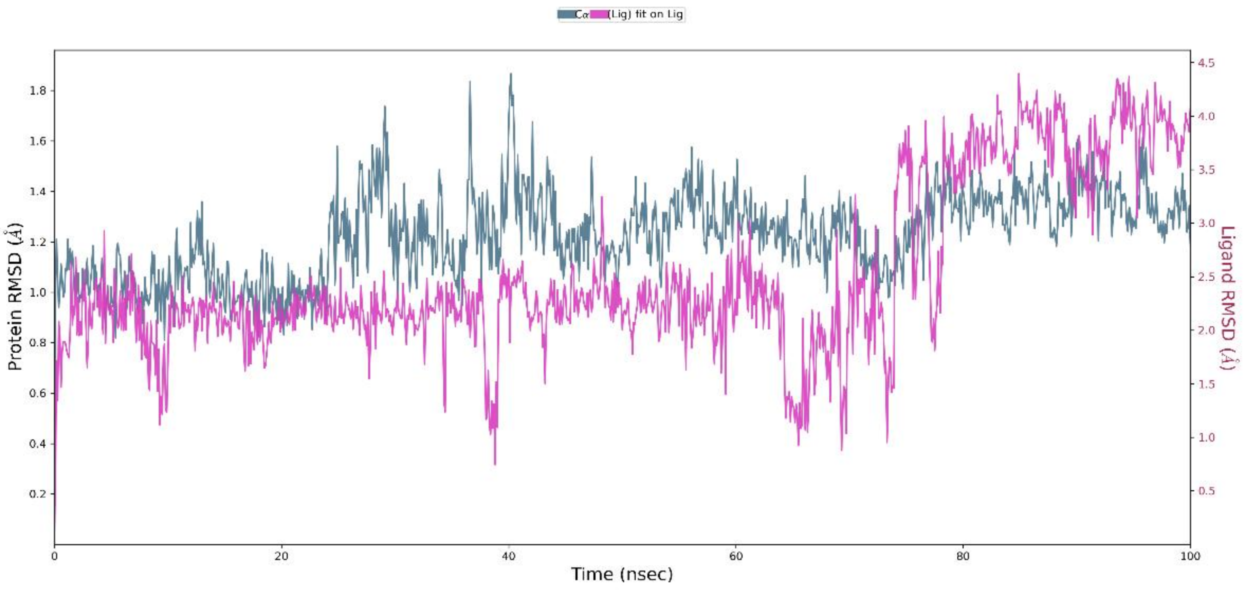

Cα (Lig) fit on Lig
Protein RMSD (Å)
Ligand RMSD (Å)
Time (nsec)

H

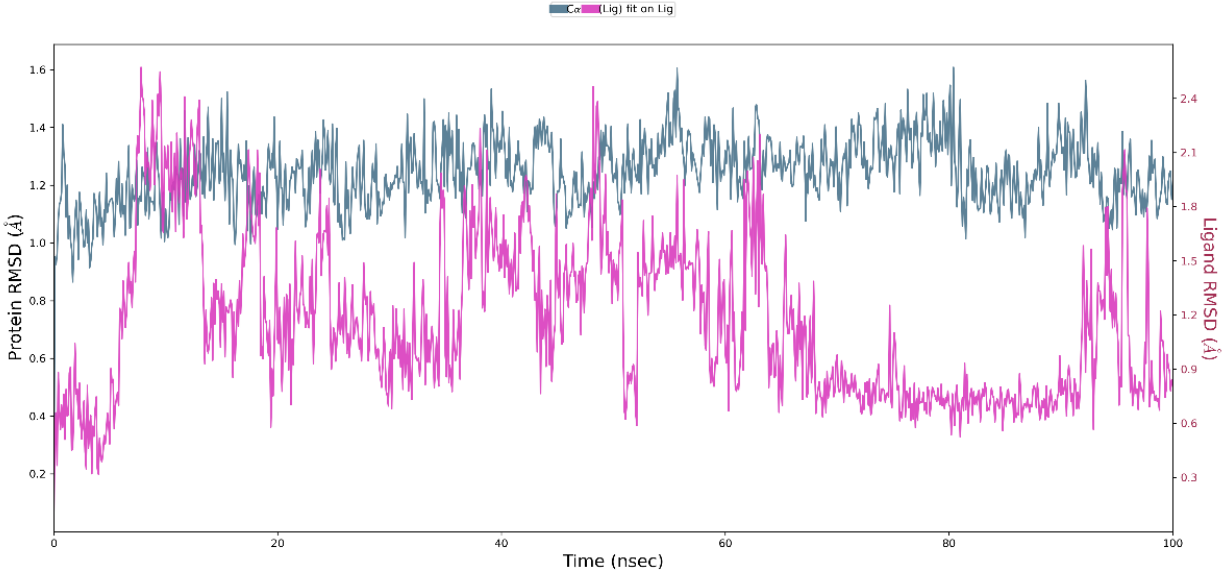

I

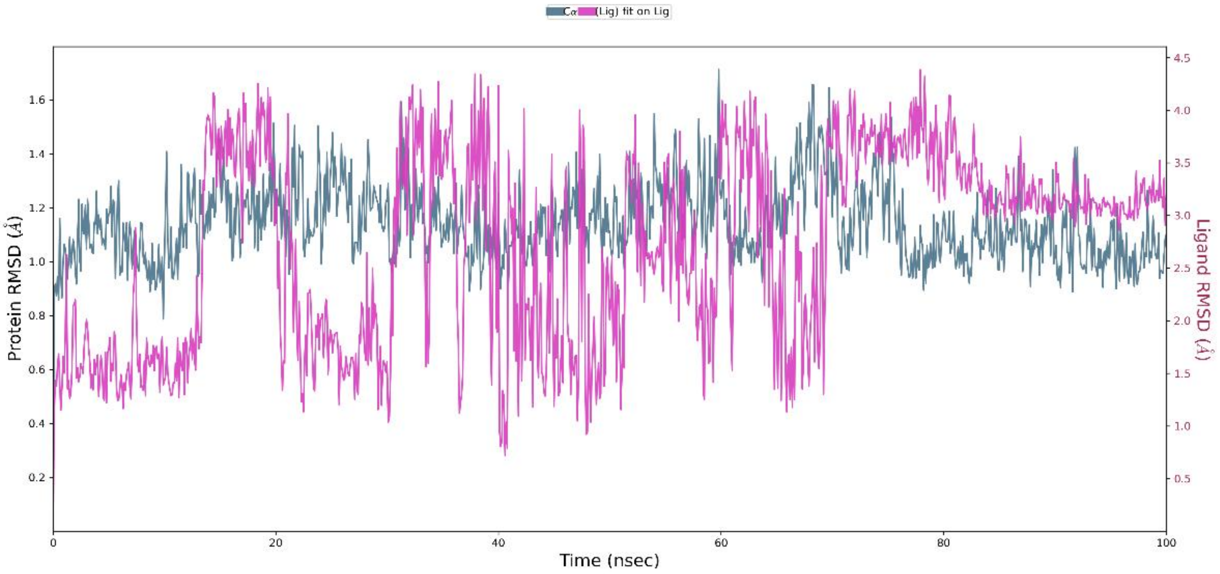

Figure S1. Root-mean-square deviation (RMSD) of the protein backbone (blue) and ligand (magenta) as a function of simulation time for the co-crystallized FXa inhibitor apixaban and the investigated ISV derivatives. Panels represent (A) apixaban; (B) ISV-M04 ; (C) ISV-M06; (D) ISV-M09; (E) ISV-M16; (F) ISV-M17; (G) ISV-M18; (H) ISV-M19; and (I) ISV-M20. The left y-axis corresponds to protein RMSD (Å), whereas the right y-axis corresponds to ligand RMSD (Å).

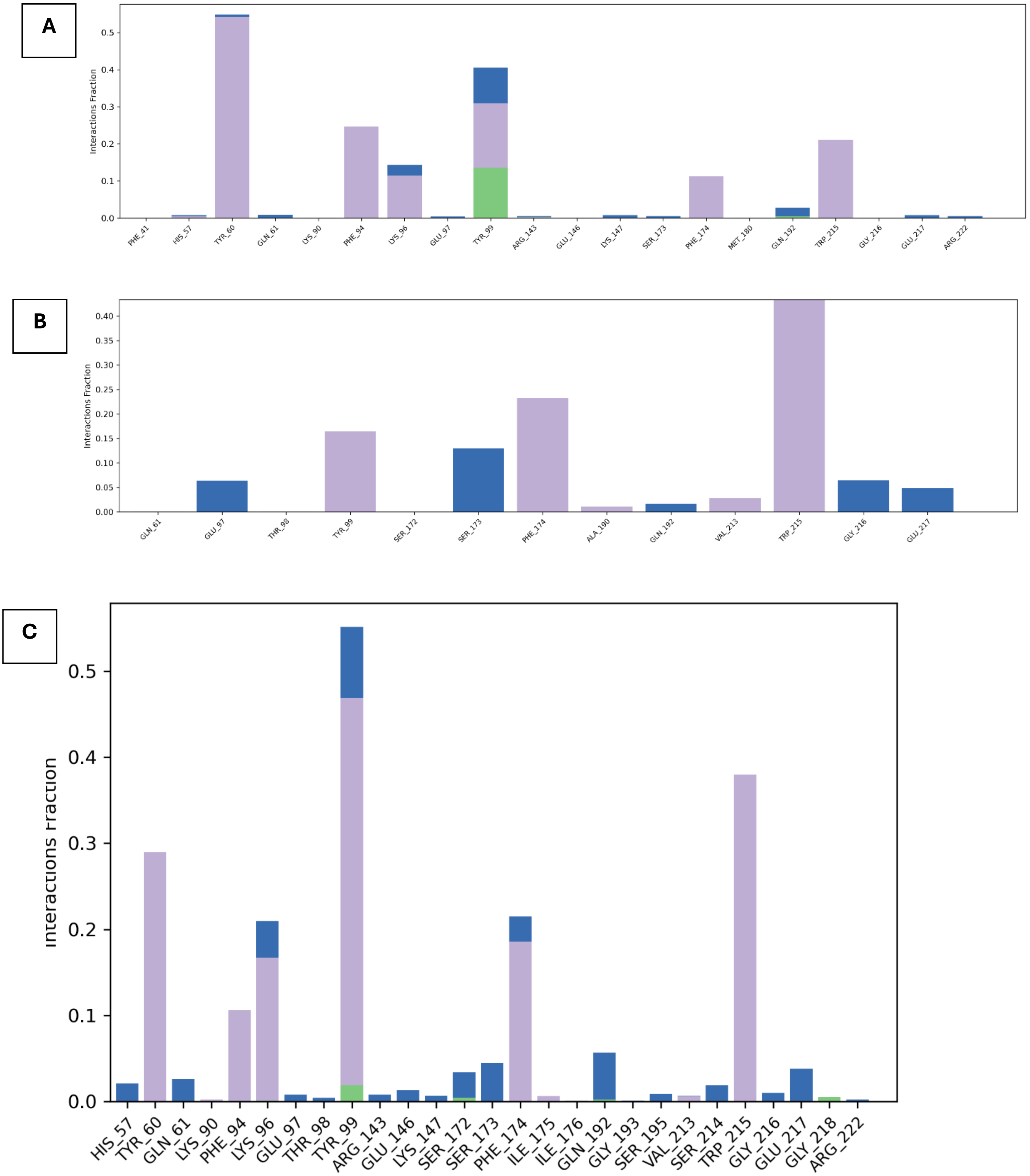
A
B
C
Interactions Fraction
PHE_41 HIS_57 TYR_60 GLN_61 LYS_90 PHE_94 LYS_96 GLU_97 TYR_99 ARG_143 GLU_146 LYS_147 SER_173 PHE_174 MET_180 GLN_192 TRP_215 GLY_216 GLU_217 ARG_222
GLN_61 GLU_97 THR_98 TYR_99 SER_172 SER_173 PHE_174 ALA_190 GLN_192 VAL_213 TRP_215 GLY_216 GLU_217
HIS_57 TYR_60 GLN_61 LYS_90 PHE_94 LYS_96 GLU_97 THR_98 TYR_99 ARG_143 GLU_146 LYS_147 SER_172 SER_173 PHE_174 ILE_175 ILE_176 GLN_192 GLY_193 SER_195 VAL_213 SER_214 TRP_215 GLY_216 GLU_217 GLY_218 ARG_222

D

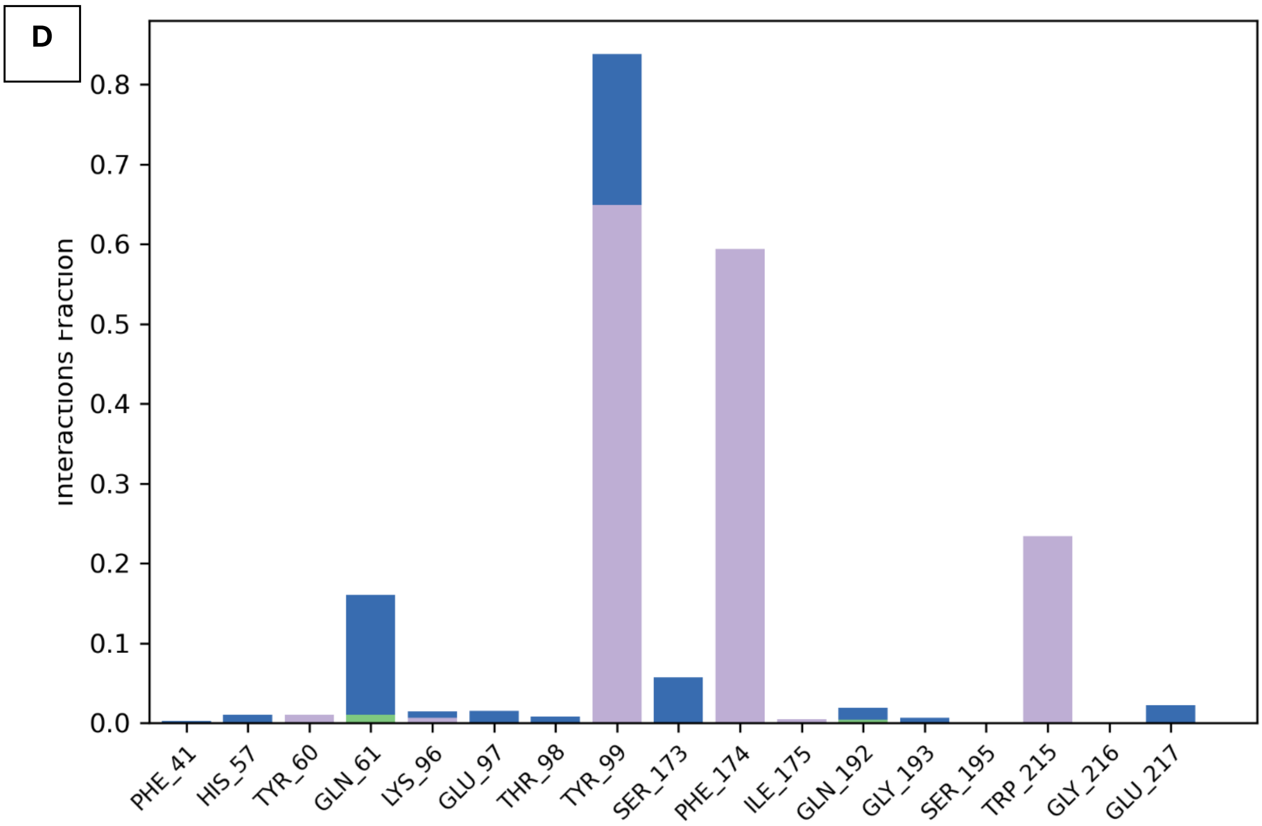
Interactions Fraction
0.8
0.7
0.6
0.5
0.4
0.3
0.2
0.1
0.0
PHE_41
HIS_57
TYR_60
GLN_61
LYS_96
GLU_97
THR_98
TYR_99
SER_173
PHE_174
ILE_175
GLN_192
GLY_193
SER_195
TRP_215
GLY_216
GLU_217

E

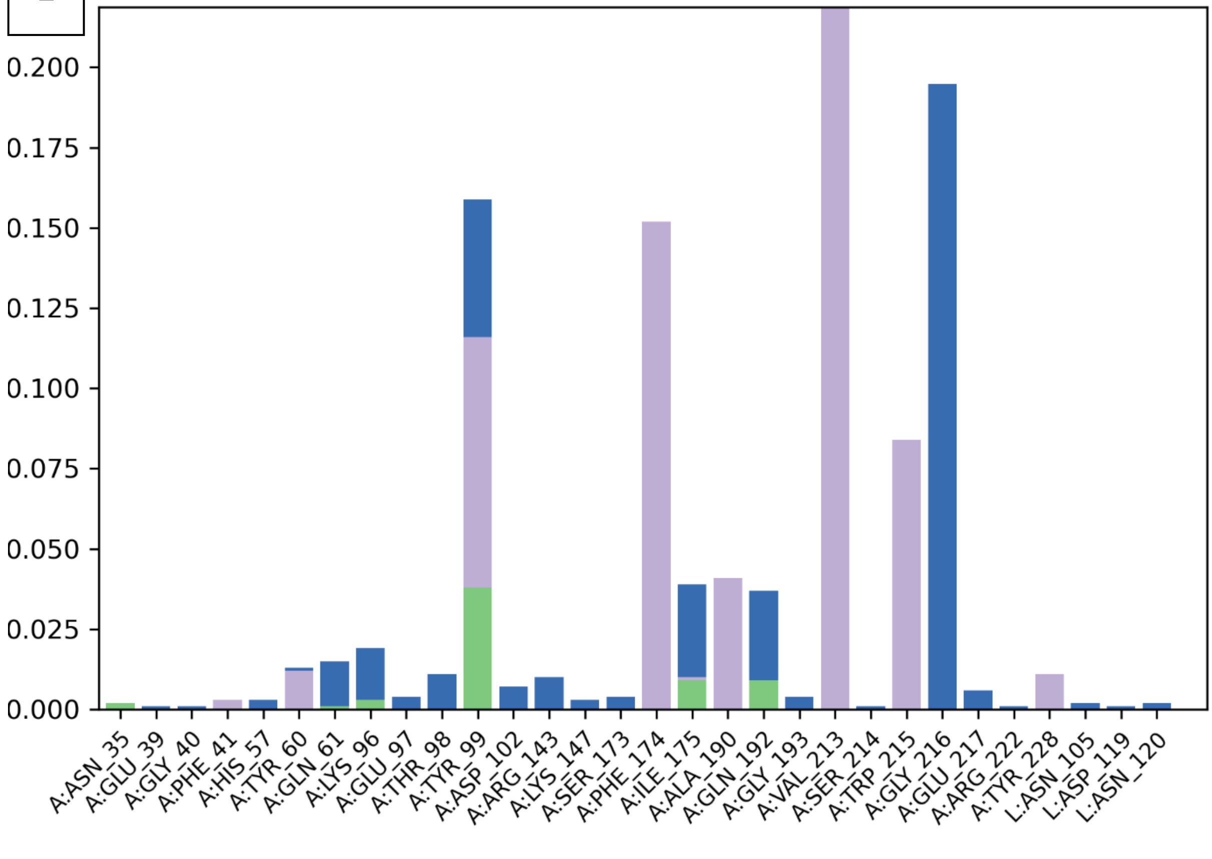
0.200
0.175
0.150
0.125
0.100
0.075
0.050
0.025
0.000
A:ASN_35
A:GLU_39
A:GLY_40
A:PHE_41
A:HIS_57
A:TYR_60
A:GLN_61
A:LYS_96
A:GLU_97
A:THR_98
A:TYR_99
A:ASP_102
A:ARG_143
A:LYS_147
A:SER_173
A:PHE_174
A:ILE_175
A:ALA_190
A:GLN_192
A:GLY_193
A:VAL_213
A:SER_214
A:TRP_215
A:GLY_216
A:GLU_217
A:ARG_222
A:TYR_228
L:ASN_105
L:ASP_119
L:ASN_120

F

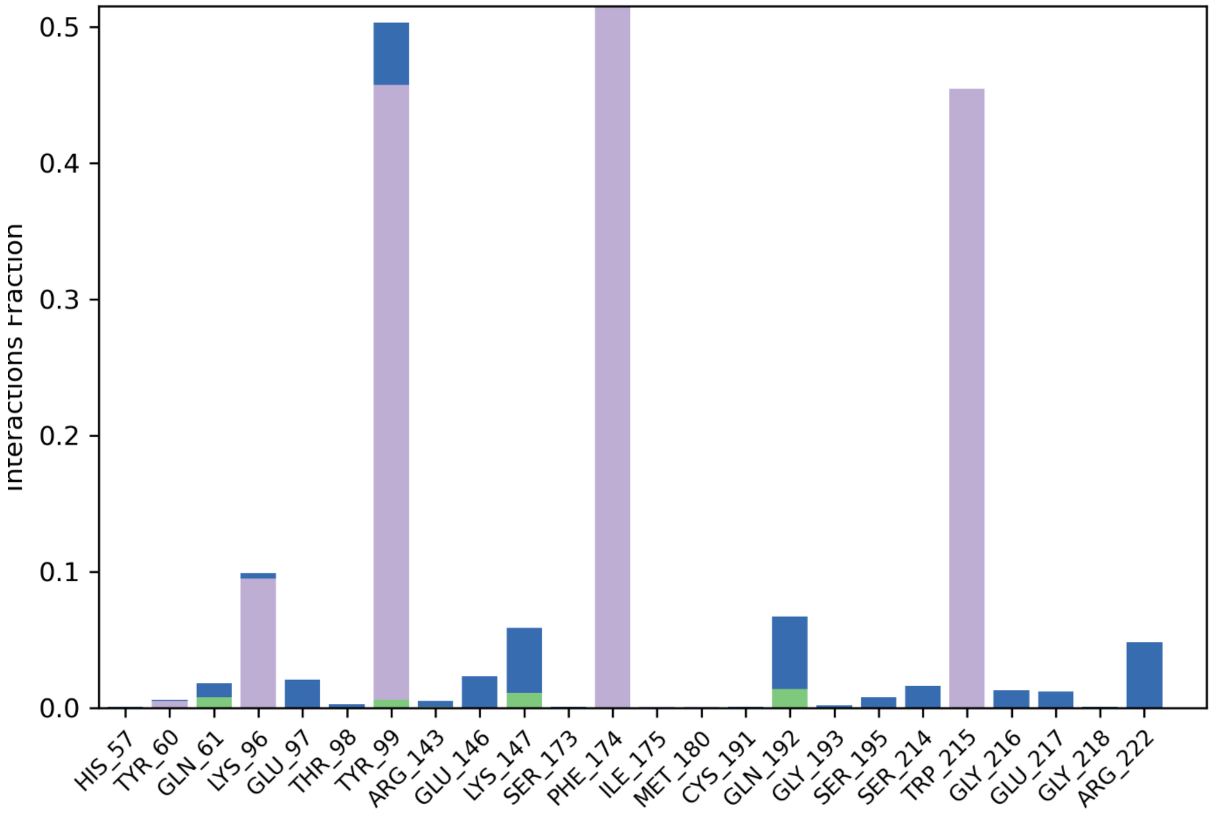

Interactions Fraction
0.0
0.1
0.2
0.3
0.4
0.5
HIS_57
TYR_60
GLN_61
LYS_96
GLU_97
THR_98
TYR_99
ARG_143
GLU_146
LYS_147
SER_173
PHE_174
ILE_175
MET_180
CYS_191
GLN_192
GLY_193
SER_195
SER_214
TRP_215
GLY_216
GLU_217
GLY_218
ARG_222

G

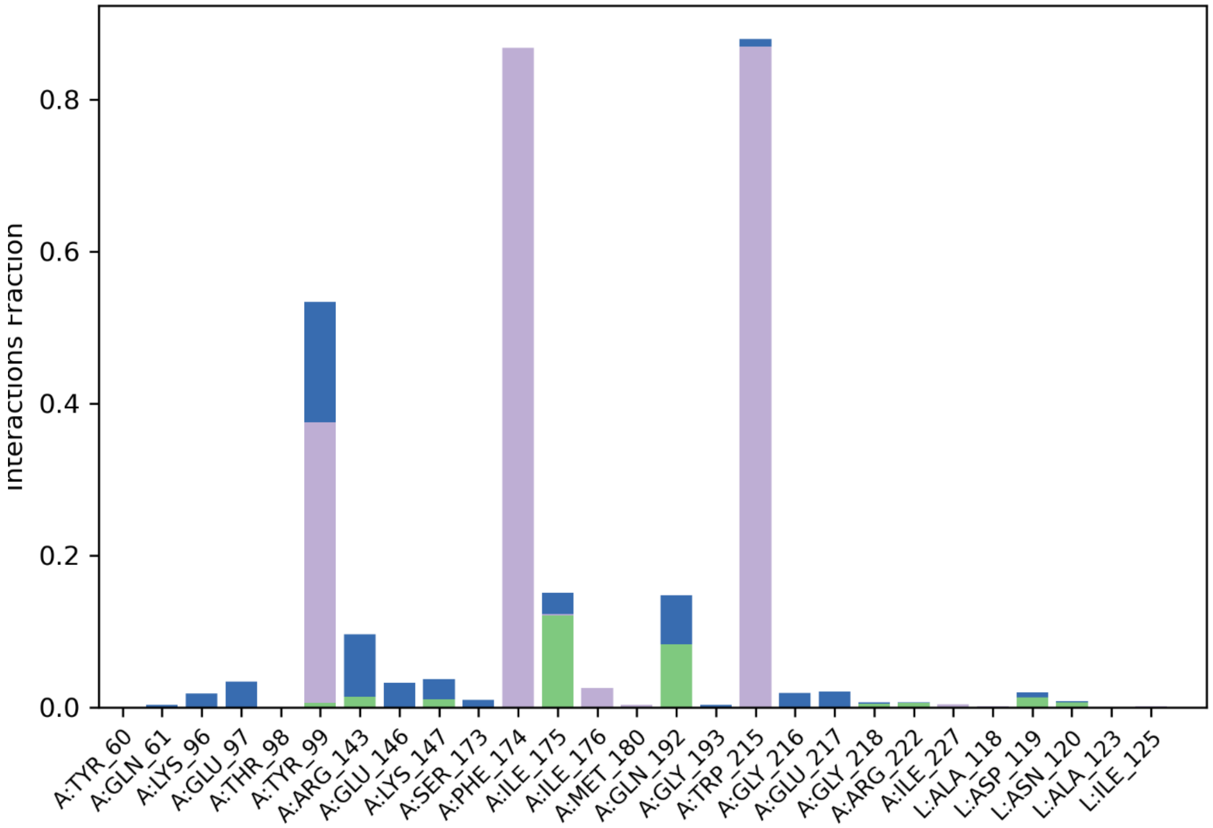

Interactions Fraction
0.0
0.2
0.4
0.6
0.8
A:TYR_60
A:GLN_61
A:LYS_96
A:GLU_97
A:THR_98
A:TYR_99
A:ARG_143
A:GLU_146
A:LYS_147
A:SER_173
A:PHE_174
A:ILE_175
A:ILE_176
A:MET_180
A:GLN_192
A:GLY_193
A:TRP_215
A:GLY_216
A:GLU_217
A:GLY_218
A:ARG_222
A:ILE_227
L:ALA_118
L:ASP_119
L:ASN_120
L:ALA_123
L:ILE_125

H

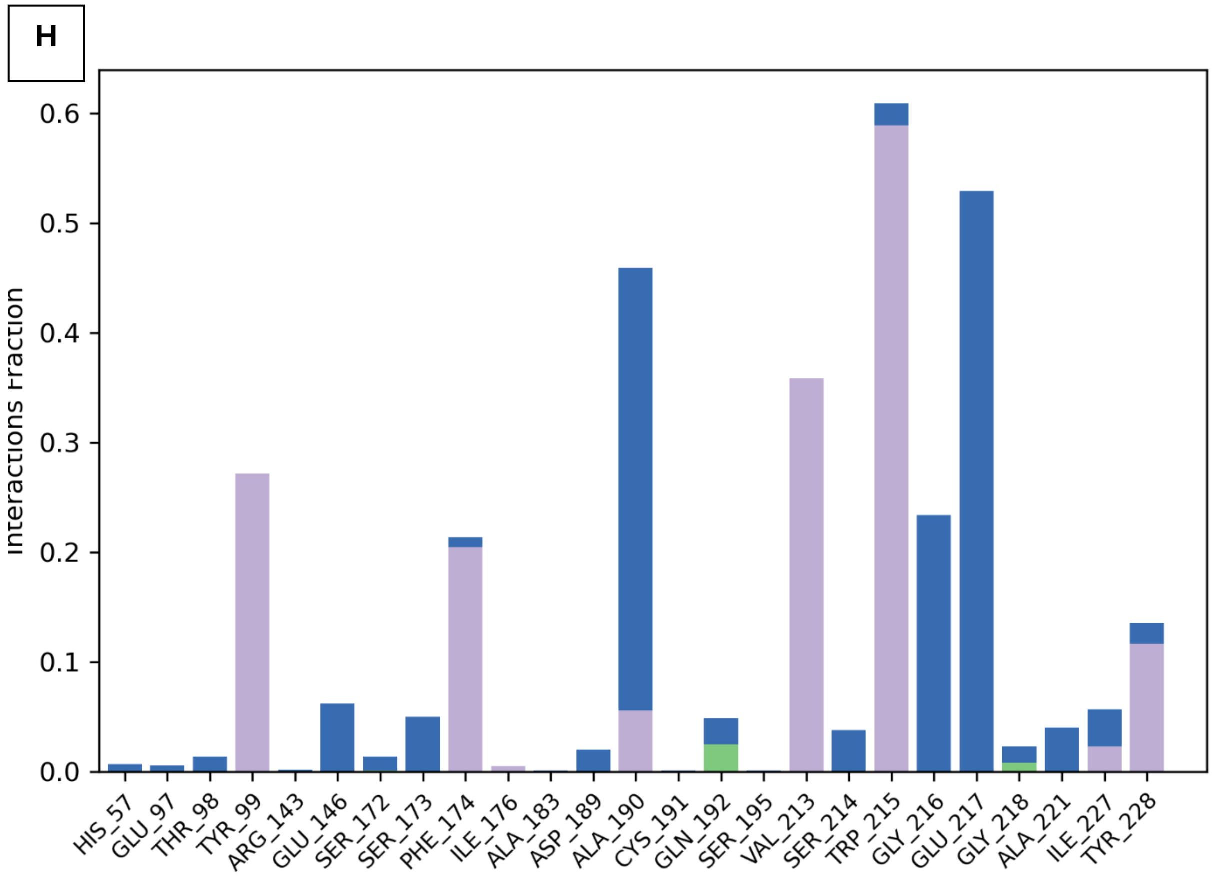

Figure S2. Protein–ligand contact analysis for the co-crystallized FXa inhibitor apixaban and the investigated ISV derivatives obtained from molecular dynamics simulations. Panels represent (A) apixaban, (B) ISV-M04, (C) ISV-M06, (D) ISV-M09, (E) ISV-M16, (F) ISV-M17, (G) ISV-M18, (H) ISV-M19, and (I) ISV-M20. The interaction fraction indicates the proportion of simulation time during which a given amino acid residue remained in contact with the ligand. Colored segments denote different interaction types, including hydrogen bonds, hydrophobic interactions, water bridges, and ionic contacts. Key residues discussed in the main text include Tyr99, Phe174, Trp215, Gly216, Asp189, and neighboring FXa active-site residues. Persistent interactions are indicative of residues involved in ligand stabilization within the active site of factor Xa.

A

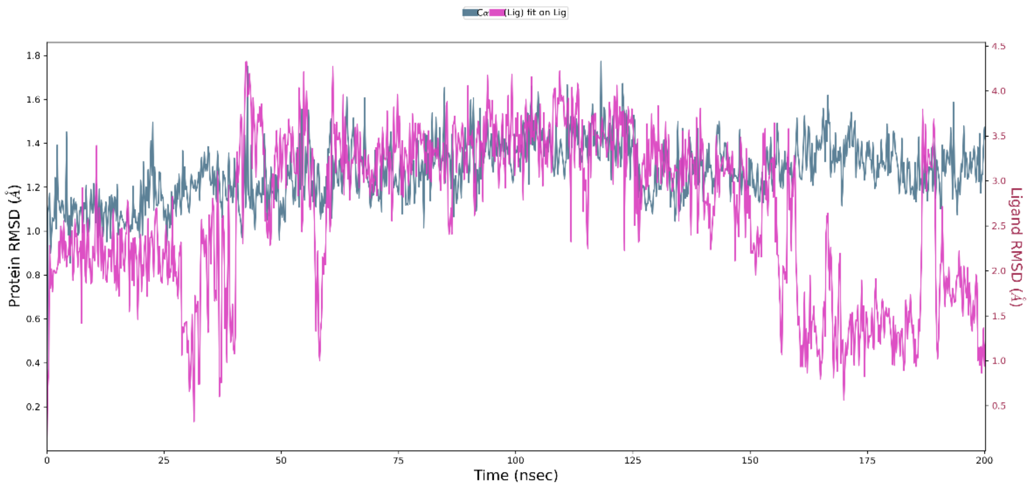

B

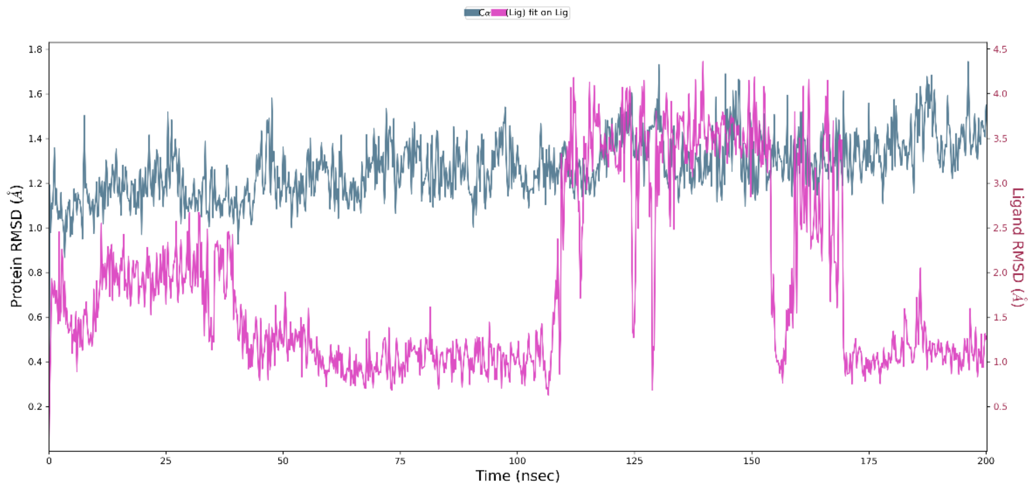

C

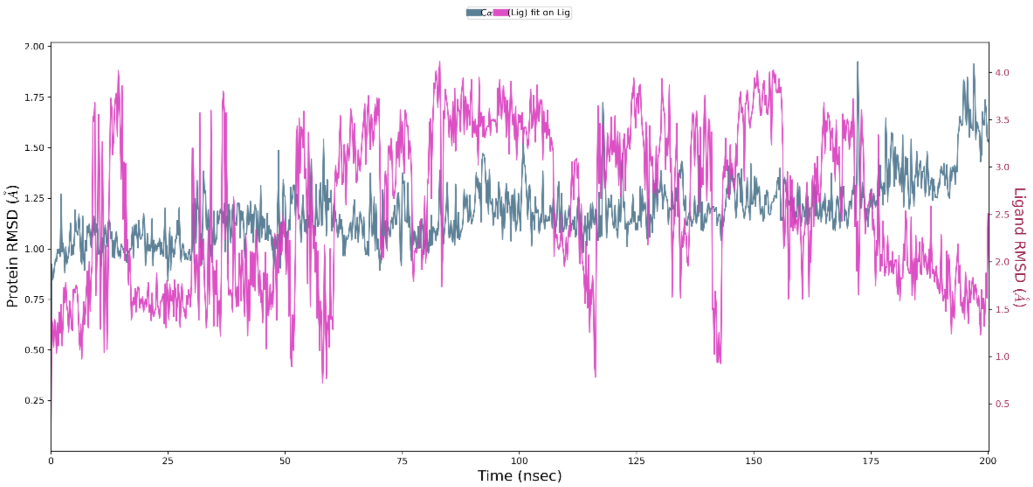

D

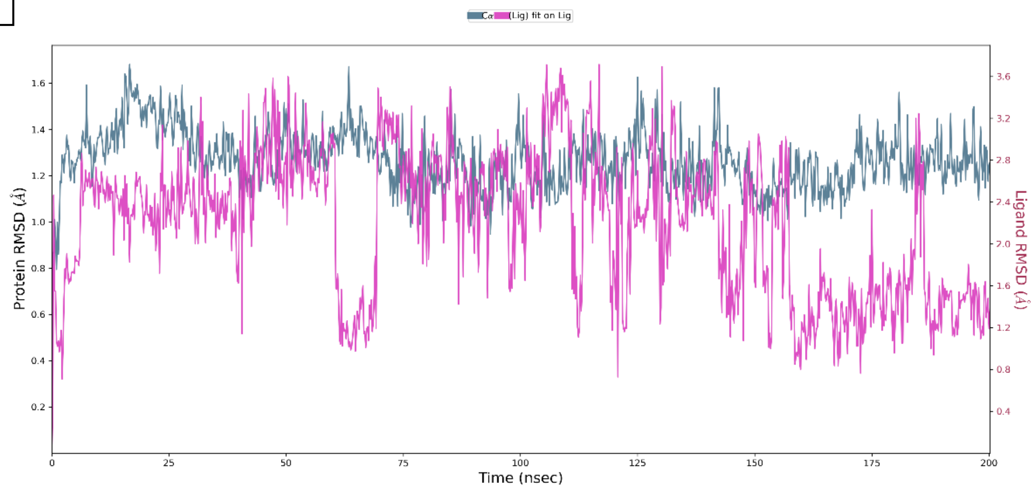

Figure S3. Root-mean-square deviation (RMSD) of the protein backbone (blue) and ligands (magenta) as a function of simulation time for the co-crystallized FXa inhibitor apixaban and the investigated ISV derivatives, extended 200 ns production MD simulations for the key Apixaban, ISV-M04, ISV-M06, and ISV-M19 complexes. Panels represent

(A) Apixaban, (B) ISV-M04, (C) ISV-M06, (D) ISV-M19. The left y-axis corresponds to protein RMSD (Å), while the right y-axis corresponds to ligand RMSD (Å)

Supplementary ADMET and Toxicity Prediction Results

The following tables summarize the SwissADME and pkCSM predictions generated for all twenty designed ISV-M01-ISV-M20 analogs. These data were used as an early-stage developability screen complementary to the QSAR, docking, molecular-dynamics, and MM/GBSA workflow.

Table S1. SwissADME physicochemical, drug-likeness, and medicinal-chemistry summary for ISV-M01-ISV-M20.

Compound	MW	TPSA	HBA/HBD	RotB	cLogP	ESOL LogS / class	GI/BBB/P-gp	Lipinski/Veber/Bioav.	PAINS/Brenk/SA
ISV-M01	551.75	76.13	5/0	7	8.51	-8.59; Poorly soluble	Low/No/No	No (2)/Yes/0.17	0/2/6.90
ISV-M02	551.75	76.13	5/0	7	8.51	-8.59; Poorly soluble	Low/No/No	No (2)/Yes/0.17	0/2/6.96
ISV-M03	568.21	76.13	4/0	7	8.74	-9.02; Poorly soluble	Low/No/Yes	No (2)/Yes/0.17	0/2/6.88
ISV-M04	568.21	76.13	4/0	7	8.74	-9.02; Poorly soluble	Low/No/Yes	No (2)/Yes/0.17	0/2/7.00
ISV-M05	547.79	76.13	4/0	7	8.49	-8.73; Poorly soluble	Low/No/No	No (2)/Yes/0.17	0/2/7.04
ISV-M06	547.79	76.13	4/0	7	8.49	-8.73; Poorly soluble	Low/No/No	No (2)/Yes/0.17	0/2/7.03
ISV-M07	563.79	85.36	5/0	8	8.07	-8.51; Poorly soluble	Low/No/Yes	No (2)/Yes/0.17	0/2/6.98
ISV-M08	533.76	76.13	4/0	7	8.13	-8.39; Poorly soluble	Low/No/No	No (2)/Yes/0.17	0/2/6.87
ISV-M09	539.79	104.37	4/0	7	8.24	-8.29; Poorly soluble	Low/No/No	No (2)/Yes/0.17	0/2/6.99
ISV-M10	534.75	89.02	5/0	7	7.52	-8.07; Poorly soluble	Low/No/No	No (2)/Yes/0.17	0/2/6.85
ISV-M11	552.74	89.02	6/0	7	7.88	-8.24; Poorly soluble	Low/No/No	No (2)/Yes/0.17	0/2/6.91

ISV-M12	548.78	89.02	5/0	7	7.87	-8.38; Poorly soluble	Low/No/No	No (2)/Yes/0.17	0/2/6.93
ISV-M13	535.69	61.03	6/0	7	7.72	-8.10; Poorly soluble	Low/No/No	No (2)/Yes/0.17	0/2/6.96
ISV-M14	531.73	61.03	5/0	7	7.70	-8.24; Poorly soluble	Low/No/No	No (2)/Yes/0.17	0/2/7.14
ISV-M15	547.72	70.26	6/0	8	7.28	-8.02; Poorly soluble	Low/No/Yes	No (2)/Yes/0.17	0/2/7.07
ISV-M16	530.74	52.82	4/0	7	7.03	-7.80; Poorly soluble	Low/No/No	No (2)/Yes/0.17	0/2/6.79
ISV-M17	548.73	52.82	5/0	7	7.29	-7.97; Poorly soluble	Low/No/No	No (2)/Yes/0.17	0/2/6.82
ISV-M18	513.66	47.89	6/0	7	7.77	-7.70; Poorly soluble	Low/No/Yes	No (2)/Yes/0.17	0/2/6.56
ISV-M19	525.69	57.12	6/0	8	7.33	-7.62; Poorly soluble	Low/No/Yes	No (2)/Yes/0.17	0/2/6.62
ISV-M20	577.74	73.19	7/0	9	8.68	-8.91; Poorly soluble	Low/No/Yes	No (2)/Yes/0.17	0/2/6.75

Abbreviations: MW, molecular weight; TPSA, topological polar surface area; HBA/HBD, hydrogen-bond acceptors/donors; RotB, rotatable bonds; cLogP, SwissADME consensus Log Po/w; GI, gastrointestinal absorption; BBB, blood-brain barrier permeation; P-gp, P-glycoprotein substrate status; Bioav., bioavailability score; SA, synthetic-accessibility score.

Table S2. pkCSM toxicity prediction summary for ISV-M01-ISV-M20.

Compound	AMES	MTD human	hERG I	hERG II	LD50 rat	LOAEL rat	Hepato.	Skin sens.	T. pyriformis	Minnow
ISV-M01	No	0.455	No	No	2.793	0.820	No	No	0.286	-2.983
ISV-M02	No	0.455	No	No	2.793	0.820	No	No	0.286	-2.983
ISV-M03	No	0.493	No	No	2.812	0.684	No	No	0.286	-3.414
ISV-M04	No	0.493	No	No	2.812	0.684	No	No	0.286	-3.414
ISV-M05	No	0.484	No	No	2.722	0.810	No	No	0.286	-3.197

ISV-M06	No	0.484	No	No	2.722	0.810	No	No	0.286	-3.197
ISV-M07	No	0.438	No	No	2.766	0.846	No	No	0.286	-3.049
ISV-M08	No	0.471	No	No	2.694	0.895	No	No	0.286	-3.218
ISV-M09	No	0.494	No	Yes	2.854	0.711	No	No	0.286	-2.768
ISV-M10	No	0.819	No	No	3.013	1.533	Yes	No	0.287	-2.989
ISV-M11	No	0.824	No	No	3.098	1.475	Yes	No	0.287	-2.773
ISV-M12	No	0.839	No	No	3.028	1.474	Yes	No	0.287	-3.085
ISV-M13	No	0.497	No	No	3.062	0.937	No	No	0.290	-2.890
ISV-M14	No	0.552	No	No	2.970	0.927	No	No	0.290	-3.104
ISV-M15	No	0.472	No	No	3.021	0.963	No	No	0.289	-2.956
ISV-M16	No	0.537	No	Yes	2.922	1.153	No	No	0.291	-3.311
ISV-M17	No	0.499	No	Yes	3.024	1.103	No	No	0.290	-3.194
ISV-M18	No	0.720	No	Yes	2.808	1.478	No	No	0.290	-1.945
ISV-M19	No	0.308	No	No	2.802	0.947	No	No	0.291	-2.111
ISV-M20	No	0.412	No	Yes	3.137	0.536	No	No	0.290	-2.548

Units: maximum tolerated dose, log mg/kg/day; oral rat acute toxicity (LD50), mol/kg; oral rat chronic toxicity (LOAEL), log mg/kg_bw/day; T. pyriformis toxicity, log ug/L; minnow toxicity, log mM.
